# Converging and Differential Brain Phospholipid Dysregulation in the Pathogenesis of Repetitive Mild Traumatic Brain Injury and Alzheimer’s Disease

**DOI:** 10.3389/fnins.2019.00103

**Published:** 2019-02-19

**Authors:** Joseph O. Ojo, Moustafa Algamal, Paige Leary, Laila Abdullah, Benoit Mouzon, James E. Evans, Michael Mullan, Fiona Crawford

**Affiliations:** ^1^Roskamp Institute, Sarasota, FL, United States; ^2^James A. Haley Veterans’ Hospital, Tampa, FL, United States; ^3^The School of Life, Health and Chemical Sciences, Open University, Milton Keynes, United Kingdom

**Keywords:** mild traumatic brain injury, Alzheimer’s disease, preclinical models, lipidomics, brain phospholipids, sphingomyelin, omega-3 fatty acid, omega-6 fatty acid

## Abstract

Repetitive mild traumatic brain injury (rmTBI) is a major epigenetic risk factor for Alzheimer’s disease (AD). The precise nature of how rmTBI leads to or precipitates AD pathology is currently unknown. Numerous neurological conditions have shown an important role for dysfunctional phospholipid metabolism as a driving factor for the pathogenesis of neurodegenerative diseases. However, the precise role in rmTBI and AD remains elusive. We hypothesized that a detailed phospholipid characterization would reveal profiles of response to injury in TBI that overlap with age-dependent changes in AD and thus provide insights into the TBI-AD relationship. We employed a lipidomic approach examining brain phospholipid profiles from mouse models of rmTBI and AD. Cortex and hippocampal tissue were collected at 24 h, 3, 6, 9, and 12 months post-rmTBI, and at ages representing ‘pre’, ‘peri’ and ‘post’ onset of amyloid pathology (i.e., 3, 9, 15 months-old). Total levels of phosphatidylcholine (PC), phosphatidylethanolamine (PE), LysoPE, and phosphatidylinositol (PI), including their monounsaturated, polyunsaturated and saturated fatty acid (FA) containing species were significantly increased at acute and/or chronic time points post-injury in both brain regions. However, levels of most phospholipid species in PS1/APP mice were nominal in the hippocampus, while in the cortex, levels were significantly decreased at ages post-onset of amyloid pathology. Sphingomyelin and LysoPC levels showed coincidental trends in our rmTBI and AD models within the hippocampus, an increase at acute and/or chronic time points examined. The ratio of arachidonic acid (omega-6 FA) to docosahexaenoic acid (omega-3 FA)-containing PE species was increased at early time points in the hippocampus of injured versus sham mice, and in PS1/APP mice there was a coincidental increase compared to wild type littermates at all time points. This study demonstrates some overlapping and diverse phospholipid profiles in rmTBI and AD models. Future studies are required to corroborate our findings in human post-mortem tissue. Investigation of secondary mechanisms triggered by aberrant downstream alterations in bioactive metabolites of these phospholipids, and their modulation at the appropriate time-windows of opportunity could help facilitate development of novel therapeutic strategies to ameliorate the neurodegenerative consequences of rmTBI or the potential triggering of AD pathogenesis by rmTBI.

## Introduction

For many years it has been known that a history of repetitive mild TBI (r-mTBI) increases the risk for the development of neurodegenerative diseases such as Alzheimer’s Disease (AD) (Gedye et al., 1989; Mortimer et al., 1991; Schofield et al., 1997; Fleminger et al., 2003; Omalu et al., 2011; McKee et al., 2013; Smith et al., 2013). Though the existence of this association is very well recognized, including the shared commonalities and distinctions between neuropathological features of TBI and AD, have long been the topic of literature reports and discussion (Gedye et al., 1989; Mortimer et al., 1991; Schofield et al., 1997; Fleminger et al., 2003; Lehman et al., 2012), the precise nature of how TBI leads to or precipitates the pathogenesis AD remains elusive. In addition, a comprehensive well-characterized timeline of molecular and pathogenic events following r-mTBI and in the pathogenesis of AD has not been studied.

Lipidomic profiling is a unique tool which enables the large-scale comprehensive analysis of novel pathways and (patho)biological networks of lipids in the CNS. The brain is one of the organs richest in lipid content and most particularly phospholipids (PL) are essential components of neuronal and non-neuronal cells in the brain. They have an integral role in the formation of membrane lipid bilayers providing structural integrity for protein function (Adibhatla and Hatcher, 2007). They can also serve as precursors for various secondary messengers that mediate signal transduction (Wenk, 2010). Fatty acids (FA) derived from PL can also be utilized as bioenergetic reservoirs, which are vital for metabolic support of neuronal function (Adibhatla and Hatcher, 2007; Wenk, 2010). Imbalances in coordination of lipid metabolism, signaling and transport, resulting in diverse phenotypes and disease states, have been well documented in several neurological and psychiatric conditions (Kosicek and Hecimovic, 2013). In AD, some studies have shown dramatic changes in PL levels such as choline plasmalogen, phosphatidylinositiol (PI) and phosphatidylethanolamine (PE), compared to age-matched controls (Stokes and Hawthorne, 1987; Nitsch et al., 1992; Prasad et al., 1998; Goodenowe et al., 2007; Igarashi et al., 2011). However, most of these findings are based on end stage AD pathology from postmortem brains. There has also been little exploration of the trajectory of lipid changes following r-mTBI in humans; to our knowledge no studies have been conducted on postmortem mTBI brains and most studies to date have been based on CSF samples collected from severe TBI patients, which show an increase in lipoprotein PL fractions and increased free fatty acids (FFA) within 48 days to 1 week after injury (Pilitsis et al., 2003; Pasvogel et al., 2010; Hankin et al., 2011). Although some of these human studies have been insightful in providing a common dysregulation of lipid involvement in TBI and AD, a prospective study examining longitudinal changes is non-existent. Moreover, the confounding heterogeneity inherent in any research of human populations, particularly the heterogeneity of etiology of human TBI can make it difficult to interpret findings derived from human studies. We propose that longitudinal studies in relevant preclinical models can assist our understanding, and so we have applied lipidomic profiling to our preclinical model of r-mTBI and an established model of AD (PSAPP). We previously described the behavioral and histopathological profiles in our established mouse model of r-mTBI from 24 h to 24 months post-injury. Compared to sham animals, injured animals demonstrate persistent deficits in spatial memory, white matter damage typified by corpus callosum thinning, axonal injury and gliosis (Mouzon et al., 2012, 2014, 2018). The [PS1(M146L), APP(K670N, M671L)] or PSAPP mouse model primarily represents amyloid-based pathology (Holcomb et al., 1998). This is a very well characterized model of AD that exhibits progressive age-dependent development of amyloid pathology and behavioral deficits in an accelerated manner (Holcomb et al., 1998; Wengenack et al., 2000; Sadowski et al., 2004; Trinchese et al., 2004). To explore the interrelationship between TBI and AD we analyzed timepoints post-TBI ranging from acute to chronic (24 h, 3, 6, 9, and 12 months) following injury at early adulthood (2–3 months) and in the PSAPP model we analyzed time points encompassing ‘pre,’ ‘peri’ and ‘post’ onset of amyloid pathology (3, 9, 15 months of age).

## Materials and Methods

### Animals

PSAPP mice expressing the PS1(M146L) mutation and the “Swedish” APP(K670N/M671L) mutation were bred in our vivarium facility on a C57BL/6 background; wild-type littermate controls were used as controls for PSAPP mice. C57BL/6 mice were acquired from Jackson Laboratories, Bar Harbor, ME, United States for breeding and for the r-mTBI/r-sham studies. Only male mice were analyzed in this study and they were randomly assigned to an N of 4 per group. PSAPP mice and their C57BL/6 littermate controls were allowed to age until euthanasia at 3, 9, and 15 months of age. Animals used for the mTBI study were 12 weeks old at the time of injury or sham injury. Mice were housed in standard cages under a 12 h (light vs. dark) schedule at ambient temperature monitored between 22° and 23°C. Animals were under specific pathogen free conditions. Animals were fed food and giving water *ad libitum* in their cages, veterinary supervision was maintained in the duration of the whole study. We did not observe any evidence of disease in the colonies used in this study. All experiments were approved by the Roskamp Institute Institutional Animal Care and Use Committee on protocol - R054. Studies were performed in accordance with Office of Laboratory Animal Welfare and National Institutes of Health guidelines.

### Experimental Closed Head Injury

The experimental closed head (mild-TBI) injury procedure was performed as we have previously described (Mouzon et al., 2012). Mice were anesthetized for 3 min with 1.5 L/min of oxygen and 3% isoflurane. Following shaving of the hair above the impact site, mice were placed in a stereotaxic frame (Just For Mice Stereotaxic, Stoelting, Wood Dale, IL, United States) fitted with an electromagnetic controlled impact device (Impact One Stereotaxic Motorized Impactor, Richmond, IL, United States). Mouse head was fixed and positioned in the stereotactic device to prevent lateral movement as the injury was administered. Body temperature was maintained at 37°C on a heating pad. Once positioned a 5-mm flat tip metal impactor attached to the electromagnetic motorized device was zeroed on the shaved and stretched scalp, directly above the midsagittal suture, and tip retracted to desired depth (1 mm) prior to the delivery of the impact using the NeuroLab controlled device (at 5 m/s and 200 ms dwell time). Animals in the TBI group received 5 TBI injuries (or sham-injury anesthesia) over a period of 9 days with an inter-injury interval of 48 h. Mice were made to recover on a heating pad (set to 37°C). This injury is sublethal and does not cause skull fracture, subdural hemorrhage or cortical damage. Animals become ambulatory after injuries and are monitored for any behavioral abnormalities.

### Lipidomic Analyses

Anesthetized animals at euthanasia were sacrificed by cardiac puncture, and perfused transcardially with phosphate buffer saline solution. Brain tissue was removed and hippocampi and cortices dissected. Cortices were homogenized in LC/MS grade water in a volume of 2.5× wet weight. Fifty microliter aliquots were stored specifically for lipidomic analysis. We followed the same methods previously described by our group (Emmerich et al., 2017). Briefly, the Folch method (Folch et al., 1957) was utilized to extract lipids from brain tissue spiked with synthetic internal standards (di-14:0 FA containing PC and PE, 14:0 FA containing (LPE) and (LPC), d18:1/17:0 SM, and di-16:0 for PI).

Lipid extracts were resuspended in isopropanol and separation was achieved by hydrophilic interaction chromatography on a 1 mm × 100 mm column packed with 3 μm Pinnacle II silica particles. We performed an isocratic run with 70% solvent A (100% acetonitrile) in 30% solvent B (78% methanol, 1% formic acid, 0.6% ammonium hydroxide) for a period of 15 min, using a flow rate of 55 μl/min, and column conditions set at 40°C. Thermo LTQ-XL linear ion trap mass spectrometer fitted with a Surveyor HPLC pumping system and Micro AS autosampler (Thermo-Fisher, Waltham, MA, United States) was used to perform mass spectrometry. In-source collision induced dissociation (SCID), with relative energies at 15%, was used to acquire full scan negative ion mass spectra from m/z 200–2,000. We obtained all generated spectra with a 200 ms maximum ion time window and by summing together 5 microscans. Mass spectra were summed over the chromatographic peak observed for each PL class and their spectra (each as a list of m/z vs. intensity) and subsequently exported to Excel. Generated excel files were subsequently uploaded to LipidomeDB for identification and quantification of each phospholipid species using the respective internal standard as a reference for each class. The mass of identified lipids species and the abundance of their isotopic variants was determined using the relevant chemical formula - tallying the masses of [M+CHO2] ions - as we have described in detail (see Emmerich et al., 2017). We utilized an independent internal reference (from one naïve mouse brain) sample to each run to control for run-to-run variability during batch processing of TBI or AD samples. All TBI samples were run together as a single batch experiment to allow all respective controls and time points to be captured in our analyses. Likewise, AD samples were also run separately with their relevant controls and age groups. All samples were run as triplicates. All molecular species identified within each phospholipid species class was summed to deduce total concentrations of PC, PE, PI, and SM species. Each phospholipid class was subsequently grouped based on their number of double bonds as an indirect measure of the degree of saturation as we have previously described (Emmerich et al., 2017).

Briefly, for each phospholipid class, we determined if their fatty acid chains contained no double bonds [as a semi-quantitative measure or indicator of saturated fatty acid (SFA) content]; if the total number of double bonds in the fatty acid chains was one [a likely indicator of monounsaturated fatty acid (MUFA) content]. Given the difficulty in determining polyunsaturated fatty acids (PUFA) containing PLs without additional fragmentation, we were unable to reliably determine their levels for the respective PLs analyzed. However, we have included a third group of classification (i.e., if the total number of double bonds in fatty acid chains were 3 or more), where PUFA containing species will be likely present. The authors acknowledge that this classification is not a definitive absolute measure of the degree of unsaturation and do not represent all SFA, MUFA and PUFA containing lipids in our samples, but an indirect measure of a significant pool. Phospholipid species containing two double bonds were excluded from these analyses given the ambiguity of double-bond position where these PL species could represent those with either two double bonds in one of fatty acid side chains or one double-bond in each fatty acid side chain. We also separately grouped arachidonic acid (AA) containing phospholipid species to docosahexaenoic acid (DHA) containing phospholipid species, and also ether (epE, ePC) and Lyso (LPE, LPC) containing phospholipid as we have described elsewhere (Emmerich et al., 2017).

### Statistical Plan

We determined differences in TBI or AD groups using ANOVA or χ2 test. Samples were log transformed when parametric assumptions were not met following tests for normality. In cases when transformation was unsatisfactory, non-parametric testing was used for analyses. We performed Principal Component Analysis (PCA) to minimize multicollinearity and to thus achieve dimension reduction for individual species relevant to each phospholipid (see Supplementary Tables T1–T19 for individual species of each phospholipid), as we have previously described for the analyses of our lipidomic datasets (Abdullah et al., 2012, 2013, 2014). Lipid species of interest were analyzed by mixed linear modeling (MLM) regression analysis to identify lipids specifically altered by the study treatment. Prior to performing MLM regression analysis on each relevant component of interest (i.e., our outcome measure), we used the Anderson-Rubin method to export uncorrelated scores, whilst adjusting for random (i.e., human) factors, and to assess independent (i.e., diagnostic and replicative) fixed factors. Following analyses using MLM, Fischer’s least significant difference (LSD) correction and the Benjamini–Hochberg (B-H) procedure were used for multiple-test correction, and control of the false discovery rate (set at 0.01) for all comparisons. Analyses was conducted using SPSS version 17 (IBM corporation) and type I error was controlled by setting *a* at 0.05.

## Results

### Phospholipid Changes in the Hippocampi of r-mTBI and AD Animal Models

#### Total Phospholipid (PE, PC, PI, and SM) Levels in the Hippocampi of r-mTBI and PSAPP Mice

Hippocampus is a region that demonstrates significant pathobiological changes in both TBI and AD pathogenesis. We therefore analyzed different total phospholipid species in this brain region of our mouse models. Total phosphatidylethanolamine (PE), phosphatidylcholine (PC) and sphingomyelin (SM) were significantly increased in the hippocampi at 24 h, 6 and 12 months post-injury in r-mTBI mice compared to shams (Table 1 and Supplementary Figures S1A,C,F). Total phosphatidylinositol (PI) levels were increased at 24 h and 12 months post-injury time points (Table 1 and Supplementary Figure S1E) compared to sham counterparts.

**Table 1:** Total phospholipid species, and arachidonic acid (AA) to decosahexaenoic acid (DHA) ratio containing phospholipids in the hippocampi of mouse models of repetitive mTBI and amyloid pathogenesis.

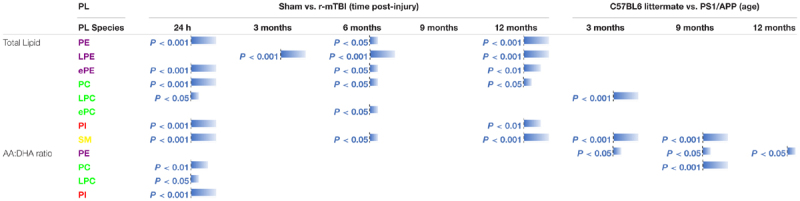

*Significant changes in total phospholipid (PE, LPE, ePE, PC, LPC, ePC, PI, SM) species, and arachidonic acid (AA) to decosahexaenoic acid (DHA) ratio containing PC, PE, and PI phospholipid species in the hippocampi of a mouse model of repetitive-mTBI (at 24 h, 3, 6, 9, and 12 months post-injury) and amyloid pathogenesis (i.e., PS1/APP mice at 3, 9, and 15 months of age). Individual molecular lipid species were quantified by liquid chromatography-mass spectrometry (LC/MS) and were generated following LipidomeDB analyses to identify total phospholipid levels/concentration, AA and DHA levels for each phospholipid species, and their corresponding ratio. Data shows *P-*values of significant changes observed following statistical analyses for comparisons between sham/r-mTBI mice or WT/PSAPP mice. Changes indicated in blue signify a significant increase in phospholipid species at a giving time point or age between sham/r-mTBI mice or WT/PSAPP mice, respectively. Raw data and concentration values per mg tissue are presented in Supplementary Figures S1–S4. Sample size for all groups across all time points is *n* = 4. Data were analyses using principal component analyses, and mixed linear modeling, followed by Fischer’s least significant difference (LSD) correction and the Benjamini–Hochberg (B–H) procedure for multiple-test correction. False discovery rate was set at 0.01. PE, Phosphatidylethanolamine; LPE, Lysophosphatidylethanolamine; PC, Phosphatidylcholine; LPC, Lysophosphatidylcholine; PI, Phosphatidylinositol; SM, Sphingomyelin; ePE, etherphosphatidylethanolamine; ePC, etherphosphatidylcholine; AA, arachidonic acid; DHA, decosahexaenoic acid.*

In WT vs. PSAPP mice, there was no change in total PE, PC or PI at any of the time points examined (Table 1 and Supplementary Figures S2A,C,E). Total SM was significantly increased at 3 and 9 months of age in PSAPP compared to WT mice (Table 1 and Supplementary Figure S2F).

#### Total Lyso-Phospholipid Species (LPE and LPC) Levels in the Hippocampi of r-mTBI and PSAPP Mice

Given the changes observed in both PE and PC species, we therefore proceeded to analyze the (Lyso) derivatives of total PE and PC in the hippocampi of our TBI and AD mouse models. Similar to changes observed above for total PE, total lysophosphatidylethanolamine (LPE) was also increased at the same timepoints at 3, 6, and 12 months post-injury compared to sham mice (Table 1 and Supplementary Figure S1B). Total phosphatidylcholine (LPC) levels were however only increased at 24 h post-injury (Table 1 and Supplementary Figure S1D) in TBI mice compared to sham counterparts.

In WT vs. PSAPP mice, there was no change in total LPE at any of the time points examined between (Table 1 and Supplementary Figure S2B), however total LPC levels was significantly increased at 3 months of age in PSAPP vs. WT mice (Table 1 and Supplementary Figure S2D), corresponding with the same changes observed in TBI mice at 24 h post-injury (i.e., 3 months of age at euthanasia).

#### Total Ether Phospholipid Species (ePE and ePC) Levels in the Hippocampi of r-mTBI and PSAPP Mice

Ether-phospholipids are also an important and physiologically relevant phospholipid species derived from total PE and PC species analyzed above. Here we also analyzed their levels in the hippocampi of both mouse models, and observed a significant increase in total ether-phosphatidylethanolamine (ePE) at 24 h, 6 and 12 months post-injury in r-mTBI mice vs. shams (Table 1 and Supplementary Figure S3A). Total ether-phosphatidylcholine (ePC) was significantly increased, only at 6 months post-injury time point in r-mTBI vs. sham mice (Table 1 and Supplementary Figure S3C). Unlike the changes observed in TBI mice, no changes was observed for ePE (Supplementary Figure S3B) or ePC (Table 1 and Supplementary Figure S3D) in PSAPP vs. WT mice at any of the age’s examined.

#### Arachidonic Acid (AA)- and Docosahexaenoic Acid (DHA)- Containing Phospholipid Species, and Their Ratio in the Hippocampi of r-mTBI and PSAPP Mice

Arachidonic acid (AA) and docosahexaenoic acid (DHA) are polyunsaturated fatty acids, with prominent pro-inflammatory and anti-inflammatory potential in the brain milieu, respectively. We thus analyzed the levels of AA to DHA containing phospholipid species (namely PE, PC, LPC, and PI) and their relative ratio in both of our models. We observed a significant increase in hippocampal AA levels for PC, PE, and PI species at 24 h and 12 months post-injury time points in r-mTBI vs. sham mice (Supplementary Figures S4A,C,E). AA levels for LPC were significantly increased at 24 h (Supplementary Figure S4G). Intriguingly, no change was observed for AA levels for PC, PE and PI species in PSAPP mice (Supplementary Figures S4B,D,E). However, there was a significant increase in AA levels for LPC at 3 months of age, and a surprisingly a significant decrease at 9 months of age (Supplementary Figure S4H).

Hippocampal DHA levels were significantly increased for PC at 24 h and 12 months post-injury, for PE species at 24 h, 6 and 12 months post-injury, for PI species at 12 months post-injury, and for LPC at 24hrs post-injury vs. sham mice (Supplementary Figures S4A,C,E,G). No change was observed for DHA levels for PC, PE, PI and LPC species in PSAPP mice at any age (Supplementary Figures S4B,D,F,H).

When we presented these changes as a ratio of AA to DHA for the different phospholipid species, no change was observed in the AA to DHA ratio for PC species following r-mTBI (Supplementary Figure S5A). However, we observed a significant increase in the AA to DHA ratio for PE, PI and LPC species at 24 h post-injury time points in r-mTBI vs. sham mice (Supplementary Figures S5C,E,G).

In WT vs. PSAPP mice, we observed a significant increase in the AA to DHA ratio for PC species at 9 of age (Table 1 and Supplementary Figure S5C), while AA to DHA ratio for PE species was significantly increased at 3, 9, and 15 months of age compared to littermate controls (Table 1 and Supplementary Figure S5D). No change was observed in the AA to DHA levels for PI and LPC species in PSAPP vs. sham mice at any age (Table 1 and Supplementary Figures S5F,H).

#### Classification of Phospholipid Species Based on Number of Double Bonds in the Hippocampi of r-mTBI and PSAPP Mice

The degree of fatty acid (FA) saturation can influence the function of FA in a variety of ways, thus we proceeded to interrogate phospholipid species with 0 double bonds (indicator of a pool of SFA containing PLs), 1 double bond (indicator of a pool of MUFA containing PLs), and 3 or more double bonds (indicator of a likely pool of PUFA containing PLs) in the hippocampi of our mouse models.

PC, SM, PE, and PI species with 1 double bond were significantly increased at 24 h and 12 months post-injury; SM and PE species with 1 double bond were also increased at 6 months post-injury in r-mTBI compared to sham mice (Table 2). No changes were observed for PC, SM, PE, and PI species with 1 double bond in the PSAPP group compared to littermate controls at all ages examined (Table 3).

**Table 2:** Classification of phospholipid species based on the number of double bonds (degree of fatty acid saturation) in the hippocampus of repetitive mTBI mice.

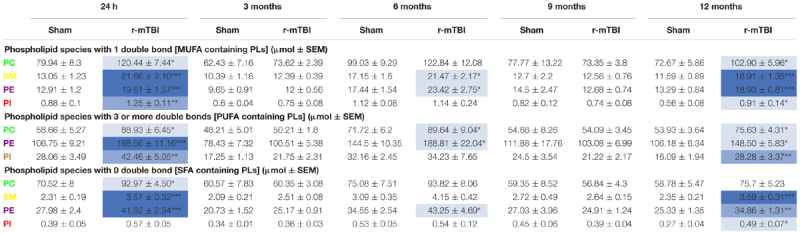

*Significant changes in PC, SM, PE, and PI species with ‘0’, ‘1’ or ‘3 or more’ double bonds in the hippocampus of repetitive mTBI mice. Sample size for all groups across all time points is *n* = 4. All data represents mean μM per (5.5 mg) wet weight ± SEM. Individual molecular lipid species were quantified by LC/MS and were summed after LipidomeDB analyses to generate degree of fatty acid classification for each phospholipid species. Blue color code indicates an increase in lipid species. Asterisks represents ^∗^*P* < 0.05 (lightest shade of blue); ^∗∗^*P* < 0.01 (middle shade of blue); ^∗∗∗^*P* < 0.001 (darkest shade of blue) for comparisons between r-mTBI/sham mice. PE, Phosphatidylethanolamine; LPE, Lysophosphatidylethanolamine; PC, Phosphatidylcholine; LPC, Lysophosphatidylcholine; PI, Phosphatidylinositol; SM, Sphingomyelin.*

**Table 3:** Classification of phospholipid species based on the number of double bonds (degree of fatty acid saturation) in the hippocampus of PSAPP mice.

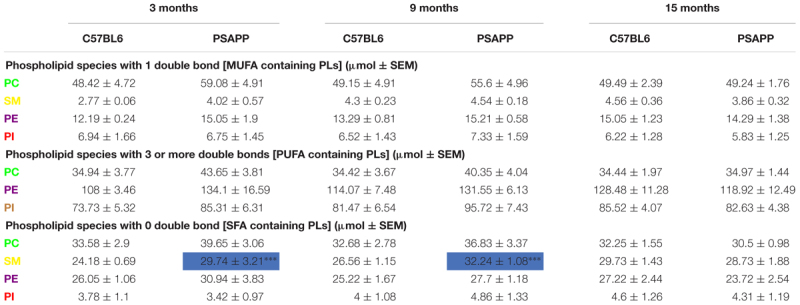

*Significant changes in PC, SM, PE, and PI species with ‘0’, ‘1,’ or ‘3 or more’ double bonds in the hippocampus of an AD mouse model. Sample size for all groups across all time points is *n* = 4. All data represents mean μM per (5.5 mg) wet weight ± SEM. Individual molecular lipid species were quantified by LC/MS and were summed after LipidomeDB analyses to generate degree of fatty acid classification for each phospholipid species. Blue color code indicates an increase in lipid species. Asterisks represents ^∗∗∗^*P* < 0.001 for comparisons between PSAPP/WT mice. PE, Phosphatidylethanolamine; LPE, Lysophosphatidylethanolamine; PC, Phosphatidylcholine; LPC, Lysophosphatidylcholine; PI, Phosphatidylinositol; SM, Sphingomyelin.*

PC, PE, and PI species with 3 or more double bonds were significantly increased at 24 h and 12 months post-injury, and PC and PE species with 3 or more double bonds were also increased at 6 months post-injury in repetitive mTBI compared to sham mice (Table 2). No changes were observed for PC, SM, PE, and PI species with 3 or more double bonds in the PSAPP group compared to littermate controls at all ages examined (Table 3).

PC, SM, and PE species with 0 double bond were significantly increased at 24 h post-injury; PE species with 0 double bond were significantly increased at 6 months post-injury; SM, PE and PI species with 0 double bond were significantly increased at 12 months post-injury in r-mTBI compared to sham mice (Table 2). SM species with 0 double bond were significantly increased in the PSAPP group at 3 and 9 months of age compared to age matched WT mice (Table 3).

See Supplementary Tables T1–T8 for changes in individual molecular species in the hippocampi for each phospholipid class in both r-mTBI and PSAPP models. Absolute concentrations values of the phospholipid species are presented in Supplementary Figures S1–S5.

### Phospholipid Changes in the Cortices of r-mTBI and AD Animal Models

We conducted similar experiments described above, on cortical tissue from our mouse model, at similar time points post-injury and age, focusing also on total phospholipid species, their lyso and ether derivatives, levels of AA to DHA containing phospholipids and their corresponding ratio, and degree of fatty acid saturation for the different phospholipids species analyzed.

#### Total Phospholipid (PE, PC, PI, and SM) Levels in the Cortices of r-mTBI and PSAPP Mice

No changes were observed in cortical total PE or SM in r-mTBI vs. sham mice (Table 4 and Supplementary Figures S6A,F). Total PC levels were significantly increased at 24 h and 12 months post-injury compared to sham counterparts (Table 4 and Supplementary Figure S6C). Total PI levels were significantly increased at 3 and 12 months post-injury compared to sham mice (Table 4 and Supplementary Figure S6E).

**Table 4:** Total phospholipid species, and arachidonic acid (AA) to decosahexaenoic acid (DHA) ratio containing phospholipids in the cortex of mouse models of repetitive mTBI and amyloid pathogenesis.

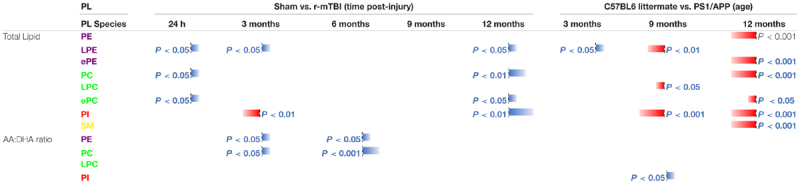

*Significant changes in total phospholipid (PE, LPE, ePE, PC, LPC, ePC, PI, SM) species, and arachidonic acid (AA) to decosahexaenoic acid (DHA) ratio containing PC, PE, and PI phospholipid species in the hippocampi of a mouse model of repetitive-mTBI (at 24 h, 3, 6, 9, and 12 months post-injury) and amyloid pathogenesis (i.e., PS1/APP mice at 3, 9, and 15 months of age). Individual molecular lipid species were quantified by LC/MS and were summed after LipidomeDB analyses to generate total phospholipid levels, AA and DHA levels for each phospholipid species, and their corresponding ratio. Data shows *P-*values of significant changes observed following statistical analyses for comparisons between sham/r-mTBI mice or WT/PSAPP mice. Changes indicated in blue signify an increase, while changes indicated in red indicate a decrease in respective phospholipid species at a giving time point or age between sham/r-mTBI mice or WT/PSAPP mice, respectively. Raw data and concentration values per mg tissue are presented in Supplementary Figures S1–S4. Sample size for all groups across all time points is *n* = 4. Data were analyses using principal component analyses, and mixed linear modeling, followed by Fischer’s least significant difference (LSD) correction and the Benjamini–Hochberg (B–H) procedure for multiple-test correction. False discovery rate was set at 0.01. PE, Phosphatidylethanolamine; LPE, Lysophosphatidylethanolamine; PC, Phosphatidylcholine; LPC, Lysophosphatidylcholine; PI, Phosphatidylinositol; SM, Sphingomyelin; ePE, etherphosphatidylethanolamine; ePC, etherphosphatidylcholine; AA, arachidonic acid; DHA, decosahexaenoic acid.*

Diverging effects were observed in the WT vs. PSAPP study, total PE, PC, and SM levels were significantly decreased at 15 months of age (Table 4 and Supplementary Figures S7A,C,F). Total PI levels were significantly decreased at 9 and also 15 months of age in PSAPP compared to littermate controls (Table 4 and Supplementary Figure S7E).

#### Total Lyso-Phospholipid Species (LPE and LPC) Levels in the Cortices of r-mTBI and PSAPP Mice

Total LPE levels were significantly increased at 24 h, 3 and 12 months post-injury compared to sham controls (Table 4 and Supplementary Figure S6B). However, no changes were observed in cortical total LPC in r-mTBI mice (Table 4 and Supplementary Figure S6D).

In the WT vs. PSAPP experimental, total LPE levels were significantly increased at 3 months and decreased at 9 months of age in PSAPP compared to littermate controls (Table 4 and Supplementary Figure S7B). However, total LPC was significantly decreased at 9 months of age in PSAPP compared to littermate controls (Table 4 and Supplementary Figure S7D).

#### Total etherPE and etherPC Levels in the Cortices of Repetitive-mTBI and PSAPP Mice

There was no significant change in ether-phosphatidylethanolamine (ePE) in r-mTBI compared to sham mice at any of the time points examined (Table 4 and Supplementary Figure S8A). We did observe an increase in total ether-phosphatidylcholine (ePC) at 24 h and 12 months post-injury in r-mTBI compared to sham mice (Table 4 and Supplementary Figure S8C). In PSAPP mice, total cortical epE and ePC levels showed differential effects, significantly decreasing at 15 months of age compared to littermate controls (Table 4 and Supplementary Figures S8B,D).

#### Arachidonic Acid and Docosahexaenoic Acid Containing Phospholipid Species, and Their Ratio in the Cortices of Repetitive mTBI and PSAPP Mice

A significant increase in cortical AA levels was observed for PC at 24 h and 12 months post-injury, and for PI species there was a significant decrease at 3 months post-injury followed by a significant increase at 12 months post-injury (Supplementary Figures S9C,E). No change was observed in AA levels for PE and LPC species at any of the time point examined post-injury (Supplementary Figures S9A,G). In the PSAPP model we observed a significant decrease at 9 and 15 months of age in AA levels for PI species (Supplementary Figure S9F). No change was observed in AA levels for PE, PC or LPC species at any age (Supplementary Figures S9B,D,H).

There was a significant increase in cortical DHA levels for PC at 24 h and 12 months post-injury in r-mTBI vs. sham mice (Supplementary Figure S9C). No significant changes were observed in DHA levels for PE, PI or LPC species in the r-mTBI/sham at any of the time points examined (Supplementary Figures S9A,E,G). In the PSAPP model there was a significant decrease in DHA levels for LPC species at 9 months of age compared to littermate controls (Supplementary Figure S9H). No changes were observed in DHA levels for PE, PC or PI in the PSAPP model at any age (Supplementary Figures S9B,D,F).

AA to DHA ratio for PE and PC species showed a significant increase at 3 and 6 months post-injury in r-mTBI compared to sham mice (Table 4 and Supplementary Figures S10A,C). However, no change was observed in the AA to DHA ratio for PE and PC in the PSAPP group compared to littermate controls at any age (Table 4 and Supplementary Figures S10B,D).

In both r-mTBI and PSAPP models there was no change in the AA to DHA ratio for PI species at any of the time points examined (Table 4 and Supplementary Figures S10E,F). For LPC species the AA to DHA ratio was unchanged in the r-mTBI model at all time points (Table 4 and Supplementary Figure S10G), while there was a significant increase in PSAPP compared to littermate controls at 9 months of age only (Table 4 and Supplementary Figure S10H).

#### Classification of Phospholipid Species Based on Number of Double Bonds in the Cortices of r-mTBI and PSAPP Mice

PC species with 1 double bond were significantly increased at 24 h and 12 months post-injury, PI species with 1 double bond were increased only at 12 months post-injury in r-mTBI compared to sham mice, and SM species with 1 double bond were significantly decreased at 3 months post-injury (Table 3). In the PSAPP vs. WT study, PI species with 1 double bond were decreased at 9 months of age (Table 5). PC, SM, PE and PI species with 1 double bond were all decreased at 15 months of age in the PSAPP group compared to age matched littermate controls (Table 6).

**Table 5:** Classification of phospholipid species based on the number of double bonds (degree of fatty acid saturation) in the cortex of repetitive mTBI mice.

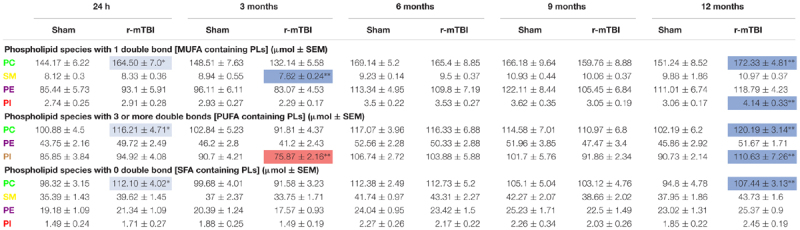

*Significant changes in PC, SM, PE, and PI species with ‘0’, ‘1’ or ‘3 or more’ double bonds in the cortex of repetitive mTBI mice. Sample size for all groups across all time points is *n* = 4. All data represents mean μM per (10 mg) wet weight ± SEM. Individual molecular lipid species were quantified by LC/MS and were summed after LipidomeDB analyses to generate degree of fatty acid classification for each phospholipid species. Blue color code indicates an increase in lipid species, while a red color code indicates a decrease in lipid species. Asterisks represents ^∗^*P* < 0.05 (lightest shade of blue or red); ^∗∗^*P* < 0.01 (middle shade of blue or red); ^∗∗∗^*P* < 0.001 (darkest shade of blue or red) for comparisons between r-mTBI/sham mice. PE, Phosphatidylethanolamine; LPE, Lysophosphatidylethanolamine; PC, Phosphatidylcholine; LPC, Lysophosphatidylcholine; PI, Phosphatidylinositol; SM, Sphingomyelin.*

**Table 6:** Classification of phospholipid species based on the number of double bonds (degree of fatty acid saturation) in the cortex of PSAPP mice.

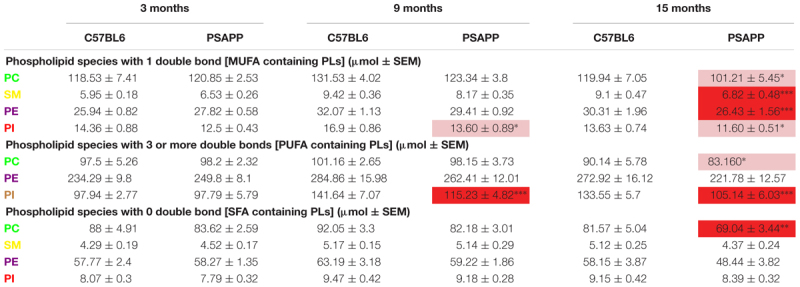

*Significant changes in PC, SM, PE, and PI species with ‘0’, ‘1’ or ‘3 or more’ double bonds in the cortex of an AD mouse model. Sample size for all groups across all time points is *n* = 4. All data represents mean μM per (10 mg) wet weight ± SEM. Individual molecular lipid species were quantified by LC/MS and were summed after LipidomeDB analyses to generate degree of fatty acid classification for each phospholipid species. Red color code indicates a decrease in lipid species. Asterisks represents ^∗^*P* < 0.05 (lightest shade of red); ^∗∗^*P* < 0.01 (middle shade of red); ^∗∗∗^*P* < 0.001 (darkest shade of red) for comparisons between PSAPP/WT mice. PE, Phosphatidylethanolamine; LPE, Lysophosphatidylethanolamine; PC, Phosphatidylcholine; LPC, Lysophosphatidylcholine; PI, Phosphatidylinositol; SM, Sphingomyelin.*

PC species with 3 or more double bonds were significantly increased at 24 h and 12 months post-injury, but decreased at 3 months and increased at 12 months post-injury for PI in r-mTBI compared to sham mice (Table 5). PC species with 3 or more double bonds were decreased at 15 months of age, with this class of phospholipids for PI also decreased at 9 and 15 months of age in the PSAPP group compared to age matched littermate controls (Table 6).

PC species with 0 double bond were significantly increased at 24 h and 12 months post-injury in r-mTBI compared to sham mice (Table 5). While PC species with 0 double bond were differentially decreased in the PSAPP group at 15 months of age compared to age matched littermate controls (Table 6).

See Supplementary Tables T9–T15 for changes in individual molecular species in the cortex for each phospholipid class in both r-mTBI and PSAPP models. Absolute concentrations values of the phospholipid species are presented in Supplementary Figures S6–S10.

## Discussion

At the outset of this project we designed a longitudinal study to comprehensively investigate changes in brain phospholipid profiles in TBI (24 h to 12 months post-injury) and AD (3–15 months of age) mouse models. Our hypothesis was that a detailed lipid characterization would reveal profiles of response to injury in our TBI model that overlap with age-dependent changes in the AD Model and thus provide insights into the TBI-AD relationship. We interrogated the hippocampus and cortex of animals, on the basis that these brain regions were anatomical substrates for the neurobehavioral deficits observed in our models. Our TBI model has been well characterized up to 24 months post injury (Mouzon et al., 2012, 2014, 2018; Ojo et al., 2015). We have demonstrated progressive deficits in hippocampal-dependent spatial learning and memory tasks, axonal injury, gliosis, and chronic changes in dendritic abnormalities and electron dense dark neurons in the cortex. In the transgenic AD model (PSAPP), mice exhibit progressive age-dependent development of amyloid pathology, astrogliosis and microgliosis in an accelerated manner (Holcomb et al., 1998; Wengenack et al., 2000; Sadowski et al., 2004; Trinchese et al., 2004), and this correlates with progressive age-related deterioration in spatial learning and working memory tasks at chronic stages (>15 months) compared to younger mice aged 5–7 months (Holcomb et al., 1999).

### Changes in PC, PE, and PI Phospholipid Species and Their Derivatives in the Neurodegenerative Sequelae of TBI and AD Pathogenesis

Our lipidomic analyses focused on six phospholipid species: phosphatidylcholine (PC), lysoPC (LPC), phosphatidylinositol (PI), phosphatidylethanolamine (PE), lysoPE (LPE), and sphingomyelin (SM). There was a significant overlap in very few brain lipid profiles in both TBI and AD models, notable and distinct differences unique to each model were mainly observed (see Tables 1, 2). We report a significant increase in PC, PE (ether-PE) and PI levels at acute (24 h) and chronic (6–12 months) time points post-injury in r-mTBI animals within the hippocampus. In the PSAPP model we observed no significant changes in these phospholipid species at any of the time points we examined. In the cortex, PC and PI species were significantly increased at both acute and subacute (3 months) or chronic time points post-injury following r-mTBI, whereas in the PSAPP model we observed a reduction in the levels of PC, PI and ether-PE at 9 and/or 15 months of age, post-onset of amyloidopathy. These findings demonstrate distinct differences in TBI and AD total phospholipid profiles, and specific brain regional responses to phospholipid metabolism.

A review of the literature reveals that, where lipid analyses have been carried out in human TBI cases, changes similar to those found in our current study have been reported. An immediate and significant increase in total phospholipid (PL) levels in CSF lipoprotein fractions from patients exposed to severe TBI was significantly correlated with neurological outcome and survival (Kay et al., 2003). In animal models of severe TBI, similar outcomes have also been demonstrated. We previously showed an increase in PC, PE and PI in the hippocampus at 3 months following a controlled cortical impact (CCI) injury (Abdullah et al., 2014). Other investigators have also shown early (after 4 days) elevations in phospholipid metabolites in the cortices of injured compared to control rats immediately following severe TBI (Homayoun et al., 1997). Our study is the only one to report longitudinal changes up to 1-year post-injury in a mouse model of r-mTBI, and we show that the immediate changes in phospholipids do not completely resolve and appear exacerbated at chronic time points post-injury. Such changes in PL levels could implicate a dysregulation in metabolic and catabolic processes for these lipid species, deficits in peroxisomal function involved in phospholipid synthesis, or a possible failure of abluminal lipid efflux transporters. In human AD cases, phospholipid levels have been previously reported to dramatically change compared to age matched controls. Significant reductions in levels of PI (68%) and PE (15%) have been reported in post-mortem (gray matter) tissue in AD compared to controls (Stokes and Hawthorne, 1987; Nitsch et al., 1992; Prasad et al., 1998) and also in our MCI/AD cases (Abdullah et al., 2017, Personal communication). Increased PL hydrolysis has been suggested as an underlying mechanism for reduced levels of PL in AD brain tissue. This is evidenced by the increase in phospholipid (PC) metabolites observed in the CSF of AD patients (Mulder et al., 2003; Walter et al., 2004). In line with these data we observed an increase in lysoPC and lysoPE (both indicative of PL metabolism) in the hippocampus and/or cortex at 3 or 9 months of age in the PSAPP model, and a reduction in PL levels in the cortex. However, there was an intriguing lack of change in the hippocampus. The consequences of PL reduction may have serious effects on the integrity of neurons, glia and cerebrovascular cell functions, and may contribute to the pathogenesis of AD, especially since PL play such a significant role in regulating the behavior of membrane proteins and enzymes involved in APP processing (Jicha and Markesbery, 2010). The reason behind the differences seen in the hippocampi and cortices of PSAPP mice is unknown. Clearly in the PSAPP model the emergence of mutagenic genotype driven amyloid pathology is present early at 6 months of age in both the hippocampus and cortex (Holcomb et al., 1998; Gordon et al., 2002), suggesting that these brain regional lipid differences are not directly linked to the onset of amyloidogenesis. No brain-region specific lipidomic characterization of phospholipids has been performed thus far in mouse models, levels of phospholipids were typically higher in the cortex compared to the hippocampus of our models, and as is the case in the human studies described above, a similar pattern also emerges, with higher levels of PLs observed in the cortex compared to the hippocampus. It thus appears that disruption of lipid homeostasis through a significant increase OR decrease in these lipid species could be detrimental to brain (patho)physiology as we have observed in our TBI and AD models. Future studies will need to explore enzymatic hydrolysis or trafficking/transport of phospholipids in these brain regions and how they impact on lipid pathways and neurodegenerative processes.

Ether phospholipids, particularly plasmalogens [epE (38:6); (40:4); (40:5); (40:6) - Supplementary Tables T2, T14, and T16] that contain O-alk-1-enyl at the sn1 position, play a role in cholesterol esterification, maintenance of myelin covered axonal nodes, and vesicular formation/exocytosis (Lohner, 1996; Gorgas et al., 2006; Mankidy et al., 2010). The increase in hippocampal ether PE levels seen in our r-mTBI model indicates a possible activation of reparative processes involving attempts at remyelination of damaged or sheared axons, and stimulation of exocytosis and neurotransmitter signaling. Whereas, in the AD model, the reduction observed could signal a significant breakdown in myelin integrity, saltatory conduction, and neurotransmission. A deficiency of plasmalogen content within phospholipids in the gray matter has been previously highlighted as a prominent feature of AD (Han et al., 2001; Gorgas et al., 2006). It thus appears that the opposite or discrepant effects in the TBI model (versus AD) may be an attempt to repair, whereas in the AD model it may signify a purely degenerative feature.

### Changes in Sphingomyelin Lipid Species in the Neurodegenerative Sequelae of TBI and AD Pathogenesis

We analyzed sphingomyelin levels in both models, and report a significant increase at acute (24 h) and chronic (6–12 months) time points in the hippocampus of injured animals, with no appreciable changes observed in the cortex. In the PSAPP model an increase in sphingomyelin in the hippocampus at 3 and 9 months of age, and a reduction in the cortex at 15 months of age was observed. These findings reflect co-incidental changes within the hippocampus in both TBI and AD models. In line with this study, we previously showed similar trends of increased SM in the hippocampus 3 months post-CCI (Abdullah et al., 2014). In AD, previous studies have investigated levels of SM with contradictory results, most reports show an increase in SM levels in human AD gray matter and cerebellum (Pettegrew et al., 2001; Bandaru et al., 2009), and a 50% increase in SM has also been reported in the CSF of prodromal AD cases (Kosicek et al., 2012). Two separate studies, however, reported a reduction in SM levels in homogenates from the frontal and temporal gyrus (Cutler et al., 2004; He et al., 2010). However, these latter studies were conducted in a smaller group of individuals (7–9) than the former studies (30–45) and therefore require further validation. The reason behind the discrepant changes observed in the cortex and hippocampus herein also remains unknown, to our knowledge no human studies to date has examined comparative levels of sphingomyelin in both brain regions in AD cases. Several outcomes can, however, be predicted from increases in sphingomyelin. It is associated with lipid rafts and is expressed in the membranous myelin sheath surrounding nerve cell axons (Kosicek and Hecimovic, 2013) thus an increase in SM levels could have an influence on membrane sorting-trafficking and remyelination of damaged axons which will be anticipated as part of the repair mechanism in our TBI model. Sphingomyelin can also be degraded by sphingomyelin synthatase 2 into diacylglycerol that has a role in the activation of protein kinase C, which is important for neuronal survival and reparative mechanisms (Kosicek and Hecimovic, 2013). Deleterious effects can also arise from the hydroxylation of sphingomyelin into ceramides, which is known to activate apoptotic signaling pathways involved in neurodegeneration (Kosicek and Hecimovic, 2013).

### Changes in the Degree of Fatty Acid Saturation of Phospholipids in the Neurodegenerative Sequelae of TBI and AD Pathogenesis

Degree of FA saturation can influence the function of FA in a variety of ways and this can impact neuronal and brain function. We classified phospholipids based on their number of double bonds as an indicator of a potential pool of SFA (0 double bond), MUFA (1 double bond), and PUFA (3 or more double bonds). As indicated in our methodology we acknowledge the limitations of this analytical approach in classifying the degree of FA saturation, particularly for the pool of PUFA containing PLs which require additional fragmentation to definitively determine their structural composition and absolute levels. Our study thus only provides a semi-quantitative measure, and thus should be interpreted with this knowledge of our limited analytical approach.

We found distinct patterns of changes in both r-mTBI and AD models, including unique regional brain differences. In the r-mTBI model levels of PC and PI fractions for all three different classifications above were significantly increased in the hippocampus and cortex at acute and/or chronic time points. Hippocampal SM containing 0 and 1 double bonds (i.e., SFA or MUFA containing) were also increased following TBI. In the AD model, we observed no changes in any of the three different classifications for PC and PI species in the hippocampus, but a notable reduction in all three classes of PC fractions, and a reduction in PE and PI fractions with 3 or more double bonds was observed in the cortex at 9 and/or 15 months of age. SM species with 1 double bond (i.e., MUFA containing) were reduced in the cortex of PSAPP mice.

Brain SFA levels can be directly regulated by synthesis in the brain or through diet and transport from the periphery. High dietary intake of SFAs such as palmitic and stearic acid have been associated with poor cognitive performance, and are high-risk factors for cardiovascular and brain related disease (Engelhart et al., 2002; Whitmer et al., 2005; Barnard et al., 2014). SFA’s such as octanoic and Myristic acids have been shown to cross the BBB to impact brain function (Spector, 1988a,b), contributing toward oxidative stress, inflammation, and impaired vascularization/BBB integrity (Pallebage-Gamarallage et al., 2010; Takechi et al., 2010a,b; Freeman and Granholm, 2012; Hsu and Kanoski, 2014). Such deleterious effects could be the consequences of increased SFA seen in our TBI model. In line with our findings, elevated levels of palmitic and stearic acids have also been reported in the brain of rats immediately following severe TBI (Homayoun et al., 1997).

The main monounsaturated FA found in the brain is oleic acid (Bazinet and Laye, 2014) which is present in high levels in Mediterranean diets, and has long been associated with protection against inflammatory and vascular pathologies (Carrillo et al., 2012). *In vivo* and *in vitro* studies have reported oleic acid’s ability to inhibit oxidative stress, drive polarization of M2 macrophage/microglia, promote neurotropic support and myelination (Medina and Tabernero, 2002; Carrillo et al., 2012). In human studies of TBI, high levels of oleic acid have been shown in the CSF 48 h after injury (Pilitsis et al., 2003). The elevations in MUFA-containing phospholipids in the brain following TBI could signal the activation of neuroprotective mechanisms, while MUFA reductions at later stages in the AD model could contribute to the cascade of neurodegenerative events following amyloid deposition. In support of this observation, a transgenic mouse model of AD [harboring the Swedish (K670N/M671L) and Indiana (V717F) APP mutations] placed on a diet enriched with high oleic acid levels showed neuroprotective effects typified by reduced BACE, increased amyloid clearance enzyme (IDE) and reduced amyloid plaques (Amtul et al., 2011).

### Profiles of Omega-3 and Omega 6 Fatty Acid Containing Levels of Phospholipids in the Neurodegenerative Sequelae of TBI and AD Pathogenesis

The two most predominant PUFAs in the brain are omega-6 arachidonic acid (AA) and omega-3 docosahexaenoic acid (DHA). AA and DHA each make up approximately 10% of the total FA within brain phospholipids (Bazinet and Laye, 2014). In this study we reveal a significant increase in the AA to DHA ratio (for PE and PI fractions) in the hippocampus at the acute time points after r-mTBI, while in the PSAPP model we observed a significant increase at all time points for PE fractions. Within the cortex a significant increase in AA:DHA (for PE and PC fractions) was observed at 3 and 6 months post-injury; while in the PSAPP model we did not observe any significant cortical changes.

The data from our r-TBI model are consistent with previously reported TBI work from our group showing a significant increase in brain AA:DHA in the CCI model at 3 months post-injury (Abdullah et al., 2014). Additionally, studies in humans examining CSF samples of patients exposed to TBI, have also demonstrated a significant increase in AA (in its free form) 1-week after insult and this was associated with severity of outcome using the Glasgow Outcome Scale (Pilitsis et al., 2003). Consistently, in AD cases, decreased DHA levels in post-mortem brains (Yuki et al., 2014) and increased blood omega-6 (relative to omega-3) levels have also been shown to be associated with increased cognitive decline (Conquer et al., 2000; Beydoun et al., 2007; Cherubini et al., 2007; Heude et al., 2003; Tully et al., 2003). In a recent study we showed that MCI/AD plasma and cortical tissue demonstrated a significant increase in AA:DHA ratio in different phospholipid species compared to healthy controls (Abdullah et al., 2017, Personal communication). It is noteworthy that our work herein has examined PE, PI, PC, LPC for DHA and AA containing phospholipids, and we have not explored DHA and AA containing phosphatidylserine (PS). PS is synthetized from PC and PE, and is the major DHA containing phospholipid in the brain (Kim et al., 2014). Thus we acknowledge the limitations of our study, as we have only looked at a small pool of DHA containing phospholipids in the brain. Nonetheless, considering the consistent changes observed in AA/DHA ratio for four different classes of phospholipids, and previous reports of glial activation/neuroinflammation in our models (Mouzon et al., 2012, 2014), we anticipate that similar changes may be observed in AA/DHA ratio for PS in the brain regions assessed. We plan in future studies to include DHA/AA ratio containing PS in our analyses, and to develop a bioactive lipid assay to measure free form of DHA, AA and other bioactive lipids.

AA and DHA are derived from long chain PUFAs. Arachidonic acid is a precursor of eicosanoids and leukotrienes that are main drivers of pro-inflammatory responses and vascular permeability (Wenk, 2010; Bazinet and Laye, 2014), while DHA, which comprises 97% of the brains omega-3 long chain PUFAs, is a precursor for end metabolites such as resolvins and protectins, which have a potent anti-inflammatory, anti-oxidant, anti-apoptotic and neurotrophic effect (Adibhatla and Hatcher, 2007; Ariel and Serhan, 2007; Bazinet and Laye, 2014). High concentrations of DHA are found in brain regions with high metabolic demands such as: the cortex, mitochondria organelles, synaptosomes fractions and synaptic vesicles. An increase in AA:DHA ratio suggests a shift toward inflammation and vascular disruption, possibly involving increased release of pro-inflammatory bioactive lipid metabolites (eicosanoids) and bioactive vascular factors (leukotrienes) (Bailes and Patel, 2014). Under such pro-inflammatory conditions, the vulnerability of long chain PUFAs to oxidative damage could increase the likelihood of a negative feedback on PUFAs, resulting in lipid peroxidation and generation of free radical species and toxic lipid byproducts such as 4-hydroxynonenal (4-HNE), Malondialdehyde (MDA) or 8-isoprostane. We are yet to investigate these possible outcomes in our TBI model, further work will be required to confirm this. Blocking enzymes such as phospholipase A_2_ and C involved in the conversion of phospholipids into arachidonic acid may offer therapeutic opportunity in TBI and AD. Moreover, supplementing DHA levels at the appropriate therapeutic window of opportunity could be beneficial in stimulating the brain’s DHA-dependent neuroprotective mechanisms. Dietary DHA supplements have been shown to alter brain phospholipid levels (Jumpsen et al., 1997), and in animal models of TBI, prophylactic treatment with DHA demonstrates neuroprotective outcomes (Mills et al., 2011) improving behavioral memory, reducing axonal injury and microglial activation (Bailes and Mills, 2010). Likewise, mice placed on an omega-3 fatty acid deficient diet display impaired recovery from CCI compared to omega-3 adequate mice (Desai et al., 2014). In AD, greater dietary intake of omega-3 fatty acids reduced amyloid plaques (Grimm et al., 2011) and demonstrate 60% less risk of developing or progressing to AD (Morris et al., 2003). The prospective Framingham and Minnesota atherosclerosis studies noted protection from AD (Schaefer et al., 2006; Beydoun et al., 2007), and less decline in verbal fluency in patients with increased blood omega-3 fatty acid levels (Beydoun et al., 2007). The source of essential PUFA in the brain is primarily through diet and production in the liver, which can then be transported to the brain through the BBB via several routes: as FFAs bound to albumin, in esterified forms within lipoproteins, or as lysophospholipids through transporters such as MFSD2A. Impairment in one or more of these transport mechanisms specific to AA or DHA PUFAs could account for the imbalance in omega-6 to omega-3 ratio *in vivo*.

## Conclusion

We have conducted a comprehensive assessment of phospholipid profiles in TBI and AD mouse models over a longitudinal time course. Our data indicate heterogeneous and overlapping outcomes in both models. We observed a coincidental increase in lysoPC and sphingomyelin levels and AA to DHA ratio in both TBI and AD models, reflecting the ongoing neuroinflammation and presenting one possible route by which TBI precipitates AD pathogenesis. However, total PE/ePE, PC and PI levels and their SFA, MUFA and potential PUFA containing species showed a trend toward increase at acute and/or chronic time points post-injury, while in the AD model, levels were either unchanged or reduced at later time points of age. A caveat of this study was the lack of direct statistical comparison between TBI and AD models, and also the missing time points/ages in the TBI vs. AD groups. The former was partly attributed to the design of our lipidomic workflow experiments, whereby we were only able to run batch samples of complete TBI vs. respective control sham injury groups at all time points post injury alone, or separately PSAPP vs. respective littermate control groups at all age groups, in a single experiment. As a result, we have conducted our statistical analyses in the same manner focusing on TBI specific changes over the five different time points, and also AD dependent changes over the three different age-groups, and looking for overlaps in the trajectory of profiles overtime.

Nonetheless, our data, still demonstrates significant contributions of lipid dysregulation to both AD and TBI pathogenesis, at least in these preclinical models, but the nature of the disruption is not consistent. We suggest that these differences to some extent derive from the progressive decline in AD compared to the anticipated coincidence of neurodegenerative and neuroreparative mechanisms in TBI sequelae. Therapeutic approaches in TBI are confounded by these sequelae and the changing nature of what to target, and when, and our data suggest that although TBI can initiate lipid changes which may contribute to, or even trigger, Alzheimer’s like pathogenesis, they also indicate the existence of unique TBI neurodegenerative mechanisms. We anticipate that this work will initiate future studies involving further dissection of the lipid biology (particularly downstream bioactive lipids) in TBI and AD pathogenesis that will help to identify the reparative versus degenerative brain responses to injury and thus enabling focused lipid-based treatment strategies.

## Author Contributions

FC and MM conceived the project. FC, JO, MA, and JE directed the project. FC, JO, MA, and LA planned the experiments in the whole study. JO, FC, LA, MA, and JE were involved in the preparation of the manuscript. FC, JO, and BM participated in the establishment of the animal models. MA performed the majority of experiments, supported by JO, PL, and LA. MA, JO, and LA participated in the analysis of the experimental data. All authors contributed to the manuscript.

## Conflict of Interest Statement

The authors declare that the research was conducted in the absence of any commercial or financial relationships that could be construed as a potential conflict of interest.

## Supplementary Material

FIGURE S1 Total Phospholipid levels in the hippocampi of r-mTBI mice. Significant changes in total phospholipid (PE, LPE, PC, LPC, PI, SM) species in the hippocampi of a mouse model of repetitive-mTBI **(A–F)**. *N* = 4 for all groups at each time point. All data represent mean μM per (5.5 mg) wet weight ± SEM. Individual molecular lipid species were quantified by LC/MS and were summed after LipidomeDB analyses to generate total phospholipid levels. Asterisks represent ^∗^*P* < 0.05; ^∗∗^*P* < 0.01; ^∗∗∗^*P* < 0.001 for comparisons between sham/r-mTBI mice. PE, Phosphatidylethanolamine; LPE, Lysophosphatidylethanolamine; PC, Phosphatidylcholine; LPC, Lysophosphatidylcholine; PI, Phosphatidylinositol; SM, Sphingomyelin.

FIGURE S2 Total Phospholipid levels in the hippocampi of PSAPP mice. Significant changes in total phospholipid (PE, LPE, PC, LPC, PI, SM) species in the hippocampi of the PSAPP mouse model compared to littermate controls **(A–F)**. *N* = 4 for all groups at each time point. All data represents mean μM per (5.5 mg) wet weight ± SEM. Individual molecular lipid species were quantified by LC/MS and were summed after LipidomeDB analyses to generate total phospholipid levels. Asterisks represent ^∗∗∗^*P* < 0.001 for comparisons between PSAPP/WT mice. PE, Phosphatidylethanolamine; LPE, Lysophosphatidylethanolamine; PC, Phosphatidylcholine; LPC, Lysophosphatidylcholine; PI, Phosphatidylinositol; SM, Sphingomyelin.

FIGURE S3 Total etherPE and ether PC levels in the hippocampus of repetitive-mTBI and PSAPP mice. Significant changes in total etherPE and etherPC lipid species in the hippocampus of a mouse model of repetitive-mTBI and AD **(A–D)**. Sample size for all groups across all time points is *n* = 4. All data represents mean μM per (5.5 mg) wet weight ± SEM. Individual molecular lipid species were quantified by LC/MS and were summed after LipidomeDB analyses to generate total etherphospholipid levels. Asterisks represents ^∗^*P* < 0.05; ^∗∗^*P* < 0.01; ^∗∗∗^*P* < 0.001 for comparisons between sham/r-mTBI mice or PSAPP/WT mice. ePE, etherphosphatidylethanolamine; ePC, etherphosphatidylcholine.

FIGURE S4 Arachidonic acid and decosahexaenoic acid containing phospholipid species in the hippocampus of repetitive mTBI and PSAPP mice. Significant changes in arachidonic acid and decosahexaenoic acid containing PC, PE and PI species in the hippocampus of a mouse model of repetitive-mTBI and AD **(A–H)**. Sample size for all groups across all time points is *n* = 4. Data represents mean μmol per wet weight (5.5 mg) ± SEM. Individual molecular lipid species were quantified by LC/MS and were summed after LipidomeDB analyses to generate arachidonic and decosahexaenoic acid levels for each phospholipid species. Asterisks represents ^∗^*P* < 0.05; ^∗∗^*P* < 0.01; ^∗∗∗^*P* < 0.001 for comparisons between sham/r-mTBI mice or WT/PSAPP mice. PE, Phosphatidylethanolamine; PC, Phosphatidylcholine; PI, Phosphatidylinositol; LPC, Lysophosphatidylcholine; AA, arachidonic acid; DHA, decosahexaenoic acid.

FIGURE S5 Arachidonic acid to decosahexaenoic acid ratio containing phospholipid species in the hippocampus of repetitive mTBI and PSAPP mice. Significant changes in arachidonic acid to decosahexaenoic acid ratio for PC, PE, and PI phospholipid species in the hippocampus of a mouse model of repetitive-mTBI and AD **(A–H)**. Sample size for all groups across all time points is *n* = 4. Data represents arbitrary value (ratio) value ± SEM. Individual molecular lipid species were quantified by LC/MS and were summed after LipidomeDB analyses to generate AA and DHA levels for each phospholipid species, and a ratio of AA to DHA was compiled from these values. Asterisks represents ^∗^*P* < 0.05; ^∗∗^*P* < 0.01; ^∗∗∗^*P* < 0.001 for comparisons between sham/r-mTBI mice or WT/PSAPP mice. PE, Phosphatidylethanolamine; PC, Phosphatidylcholine; PI, Phosphatidylinositol; LPC, Lysophosphatidylcholine; AA, arachidonic acid; DHA, decosahexaenoic acid.

FIGURE S6 Total Phospholipid levels in the cortex of r-mTBI mice. Significant changes in total phospholipid (PE, LPE, PC, LPC, PI, SM) species in the cortex of a mouse model of repetitive-mTBI **(A–F)**. Sample size for all groups across all time points is *n* = 4. All data represents mean μM per (10) wet weight ± SEM. Individual molecular lipid species were quantified by liquid chromatography/mass spectrometry and were summed after LipidomeDB analyses to generate total phospholipid levels. Asterisks represents ^∗^*P* < 0.05; ^∗∗^*P* < 0.01; ^∗∗∗^*P* < 0.001 for comparisons between sham/r-mTBI mice. PE, Phosphatidylethanolamine; LPE, Lysophosphatidylethanolamine; PC, Phosphatidylcholine; LPC, Lysophosphatidylcholine; PI, Phosphatidylinositol; SM, Sphingomyelin.

FIGURE S7 Total Phospholipid levels in the cortex of PSAPP mice. Significant changes in total phospholipid (PE, LPE, PC, LPC, PI, SM) species in the cortex of a PSAPP mouse model **(A–F)**. Sample size for all groups across all time points is *n* = 4. All data represents mean μM per (10 mg) wet weight ± SEM. Individual molecular lipid species were quantified by liquid chromatography/mass spectrometry and were summed after LipidomeDB analyses to generate total phospholipid levels. Asterisks represents ^∗∗∗^*P* < 0.001 for comparisons between PSAPP/WT mice. PE, Phosphatidylethanolamine; LPE, Lysophosphatidylethanolamine; PC, Phosphatidylcholine; LPC, Lysophosphatidylcholine; PI, Phosphatidylinositol; SM, Sphingomyelin.

FIGURE S8 Total etherPE and ether PC levels in the cortex of repetitive mTBI and PSAPP mice **(A–D)**. Significant changes in total etherPE and etherPC lipid species in the cortex of a mouse model of repetitive-mTBI and AD. Sample size for all groups across all time points is *n* = 4. All data represents mean μM per (10 mg) wet weight ± SEM. Individual molecular lipid species were quantified by liquid chromatography/mass spectrometry and were summed after LipidomeDB analyses to generate total etherphospholipid levels. Asterisks represents ^∗^*P* < 0.05; ^∗∗∗^*P* < 0.001 for comparisons between sham/r-mTBI mice or PSAPP/WT mice. ePE, etherphosphatidylethanolamine; ePC, etherphosphatidylcholine.

FIGURE S9 Arachidonic acid and decosahexaenoic acid containing phospholipid species in the cortex of repetitive mTBI and PSAPP mice. Significant changes in arachidonic acid and decosahexaenoic acid containing PC **(C,D)**, PE **(A,B)**, PI **(E,F)**, and LPC **(G,H)** species in the cortex of a mouse model of repetitive-mTBI and AD. Sample size for all groups across all time points is *n* = 4. Data represents mean μmol per wet weight (10 mg) ± SEM. Individual molecular lipid species were quantified by liquid chromatography/mass spectrometry and were summed after LipidomeDB analyses to generate arachidonic and decosahexaenoic acid levels for each phospholipid species. Asterisks represents ^∗∗^*P* < 0.01 for comparisons between sham/r-mTBI mice or WT/PSAPP mice. PE, Phosphatidylethanolamine; PC, Phosphatidylcholine; PI, Phosphatidylinositol; LPC, Lysophosphatidylcholine; AA, arachidonic acid; DHA, decosahexaenoic acid.

FIGURE S10 Arachidonic acid to decosahexaenoic acid ratio containing phospholipid species in the cortex of repetitive mTBI and PSAPP mice. Significant changes in arachidonic acid to decosahexaenoic acid ratio for PC **(C,D)**, PE **(A,B)**, PI **(E,F)**, and LPC **(G,H)** phospholipid species in the cortex of a mouse model of repetitive-mTBI and AD. Sample size for all groups across all time points is *n* = 4. Data represents arbitrary value (ratio) value ± SEM. Individual molecular lipid species were quantified by liquid chromatography/mass spectrometry and were summed after LipidomeDB analyses to generate AA and DHA levels for each phospholipid species, and a ratio of AA to DHA was compiled from these values. Asterisks represents ^∗^*P* < 0.05; ^∗∗∗^*P* < 0.001 for comparisons between sham/r-mTBI mice or WT/PSAPP mice. PE, Phosphatidylethanolamine; PC, Phosphatidylcholine; PI, Phosphatidylinositol; LPC, Lysophosphatidylcholine; AA, arachidonic acid; DHA, decosahexaenoic acid.

TABLE 1 Phosphatidylcholine lipid species in the hippocampus of r-mTBI mice. Significant changes in individual species containing phosphatidylcholine. Sample size for all groups across all time points is *n* = 4. All data represents mean μM per (5.5 mg) wet weight ± SEM. Individual molecular lipid species were quantified by liquid chromatography/mass spectrometry. Highlighted boxes show significantly regulated levels (*P* < 0.001) between repetitive-mTBI/sham mice based on mixed linear modeling regression analysis.

TABLE 2 Phosphatidylethanolamine lipid species in the hippocampus of r-mTBI mice. Significant changes in individual species containing phosphatidylethanolamine. Sample size for all groups across all time points is *n* = 4. All data represents mean μM per (5.5 mg) wet weight ± SEM. Individual molecular lipid species were quantified by liquid chromatography/mass spectrometry. Highlighted boxes show significantly regulated levels (*P* < 0.001) between repetitive-mTBI/sham mice based on mixed linear modeling regression analysis.

TABLE 3 Lysophosphatidylethanolamine lipid species in the hippocampus of r-mTBI mice. Significant changes in individual species containing lysophosphatidylethanolamine. Sample size for all groups across all time points is *n* = 4. All data represents mean μM per (5.5 mg) wet weight ± SEM. Individual molecular lipid species were quantified by liquid chromatography/mass spectrometry. Highlighted boxes show significantly regulated levels (*P* < 0.001) between repetitive-mTBI/sham mice based on mixed linear modeling regression analysis.

TABLE 4 Phosphatidylinositol lipid species in the hippocampus of r-mTBI mice. Significant changes in individual species containing phosphatidylinositiol. Sample size for all groups across all time points is *n* = 4. All data represents mean μM per (5.5 mg) wet weight ± SEM. Individual molecular lipid species were quantified by liquid chromatography/mass spectrometry. Highlighted boxes show significantly regulated levels (*P* < 0.001) between repetitive-mTBI/sham mice based on mixed linear modeling regression analysis.

TABLE 5 Sphingomyelin lipid species in the hippocampus of r-mTBI mice. Significant changes in individual species containing sphingomyelin. Sample size for all groups across all time points is n = 4. All data represents mean μM per (5.5 mg) wet weight ± SEM. Individual molecular lipid species were quantified by liquid chromatography/mass spectrometry. Highlighted boxes show significantly regulated levels (*P* < 0.001) between repetitive-mTBI/sham mice based on mixed linear modeling regression analysis.

TABLE 6 Lysophosphatidylcholine lipid species in the hippocampus of r-mTBI mice. Significant changes in individual species containing lysophosphatidylcholine. Sample size for all groups across all time points is *n* = 4. All data represents mean μM per (5.5 mg) wet weight ± SEM. Individual molecular lipid species were quantified by liquid chromatography/mass spectrometry. Highlighted boxes show significantly regulated levels (*P* < 0.001) between repetitive-mTBI/sham mice based on mixed linear modeling regression analysis.

TABLE 7 Sphingomyelin lipid species in the hippocampus of PSAPP mice. Significant changes in individual species containing sphingomyelin. Sample size for all groups across all time points is *n* = 4. All data represents mean μM per (5.5 mg) wet weight ± SEM. Individual molecular lipid species were quantified by liquid chromatography/mass spectrometry. Highlighted boxes show significantly regulated levels (*P* < 0.001) between PSAPP/WT mice based on mixed linear modeling regression analysis.

TABLE 8 Lysophosphatidylcholine lipid species in the hippocampus of PSAPP mice. Significant changes in individual species containing lysophosphatidylcholine. Sample size for all groups across all time points is *n* = 4. All data represents mean μM per (5.5 mg) wet weight ± SEM. Individual molecular lipid species were quantified by liquid chromatography/mass spectrometry. Highlighted boxes show significantly regulated levels (*P* < 0.001) between PSAPP/WT mice based on mixed linear modeling regression analysis.

TABLE 9 Phosphatidylcholine lipid species in the cortex of r-mTBI mice. Significant changes in individual species containing phosphatidylcholine. Sample size for all groups across all time points is *n* = 4. All data represents mean μM per (10 mg) wet weight ± SEM. Individual molecular lipid species were quantified by liquid chromatography/mass spectrometry. Highlighted boxes show significantly regulated levels (*P* < 0.001) between repetitive-mTBI/sham mice based on mixed linear modeling regression analysis.

TABLE 10 Lysophosphatidylethanolamine lipid species in the cortex of r-mTBI mice. Significant changes in individual species containing lysophosphatidylethanolamine. Sample size for all groups across all time points is *n* = 4. All data represents mean μM per (10 mg) wet weight ± SEM. Individual molecular lipid species were quantified by liquid chromatography/mass spectrometry. Highlighted boxes show significantly regulated levels (*P* < 0.001) between repetitive-mTB/sham mice based on mixed linear modeling regression analysis.

TABLE 11 Phosphatidylinositol lipid species in the cortex of r-mTBI mice. Significant changes in individual species containing phosphatidylinositol. Sample size for all groups across all time points is *n* = 4. All data represents mean μM per (10 mg) wet weight ± SEM. Individual molecular lipid species were quantified by liquid chromatography/mass spectrometry. Highlighted boxes show significantly regulated levels (*P* < 0.001) between repetitive-mTBI/sham mice based on mixed linear modeling regression analysis.

TABLE 12 Sphingomyelin lipid species in the cortex of r-mTBI mice. Significant changes in individual species containing sphingomyelin. Sample size for all groups across all time points is *n* = 4. All data represents mean μM per (10 mg) wet weight ± SEM. Individual molecular lipid species were quantified by liquid chromatography/mass spectrometry. Highlighted boxes show significantly regulated levels (*P* < 0.001) between repetitive-mTBI/sham mice based on mixed linear modeling regression analysis.

TABLE 13 Lysophosphatidycholine lipid species in the cortex of r-mTBI mice. Significant changes in individual species containing lysophosphatidylcholine. Sample size for all groups across all time points is *n* = 4. All data represents mean μM per (10 mg) wet weight ± SEM. Individual molecular lipid species were quantified by liquid chromatography/mass spectrometry. Highlighted boxes show significantly regulated levels (*P* < 0.001) between repetitive-mTBI/sham mice based on mixed linear modeling regression analysis.

TABLE 14 Phosphatidylethanolamine lipid in the cortex of PSAPP mice. Significant changes in individual species containing phosphatidylethanolamine. Sample size for all groups across all time points is *n* = 4. All data represents mean μM per (10 mg) wet weight ± SEM. Individual molecular lipid species were quantified by liquid chromatography/mass spectrometry. Highlighted boxes show significantly regulated levels (*P* < 0.001) between PSAPP/WT mice based on mixed linear modeling regression analysis.

TABLE 15 Phosphatidylcholine lipid species in the cortex of PSAPP mice. Significant changes in individual species containing phosphatidylcholine. Sample size for all groups across all time points is *n* = 4. All data represents mean μM per (10 mg) wet weight ± SEM. Individual molecular lipid species were quantified by liquid chromatography/mass spectrometry. Highlighted boxes show significantly regulated levels (*P* < 0.001) between PSAPP/WT mice based on mixed linear modeling regression analysis.

TABLE 16 Phosphatidylethanolamine lipid species in the cortex of PSAPP mice. Significant changes in individual species containing phosphatidylethanolamine. Sample size for all groups across all time points is *n* = 4. All data represents mean μM per (10 mg) wet weight ± SEM. Individual molecular lipid species were quantified by liquid chromatography/mass spectrometry. Highlighted boxes show significantly regulated levels (*P* < 0.001) between PSAPP/WT mice based on mixed linear modeling regression analysis.

TABLE 17 Phosphatidylinositol lipid species in the cortex of PSAPP mice. Significant changes in individual species containing phosphatidylinositol. Sample size for all groups across all time points is *n* = 4. All data represents mean μM per (10 mg) wet weight ± SEM. Individual molecular lipid species were quantified by liquid chromatography/mass spectrometry. Highlighted boxes show significantly regulated levels (*P* < 0.001) between PSAPP/WT mice based on mixed linear modeling regression analysis.

TABLE 18 Sphingomyelin lipid species in the cortex of PSAPP mice. Significant changes in individual species containing sphingomyelin. Sample size for all groups across all time points is *n* = 4. All data represents mean μM per (10 mg) wet weight ± SEM. Individual molecular lipid species were quantified by liquid chromatography/mass spectrometry. Highlighted boxes show significantly regulated levels (*P* < 0.001) between PSAPP/WT mice based on mixed linear modeling regression analysis.

TABLE 19 Lysophosphatidylcholine lipid species in the cortex of PSAPP mice. Significant changes in individual species containing lysophosphatidylcholine. Sample size for all groups across all time points is *n* = 4. All data represents mean μM per (10 mg) wet weight ± SEM. Individual molecular lipid species were quantified by liquid chromatography/mass spectrometry. Highlighted boxes show significantly regulated levels (*P* < 0.001) between PSAPP/WT mice based on mixed linear modeling regression analysis.
