# Supplementary figures and images for: Converging and Differential Brain Phospholipid Dysregulation in the Pathogenesis of Repetitive Mild Traumatic Brain Injury and Alzheimer’s Disease
Source: Front Neurosci. 2019 Feb 19;13:103. doi: 10.3389/fnins.2019.00103 (PMC6390207; doi:10.3389/fnins.2019.00103)

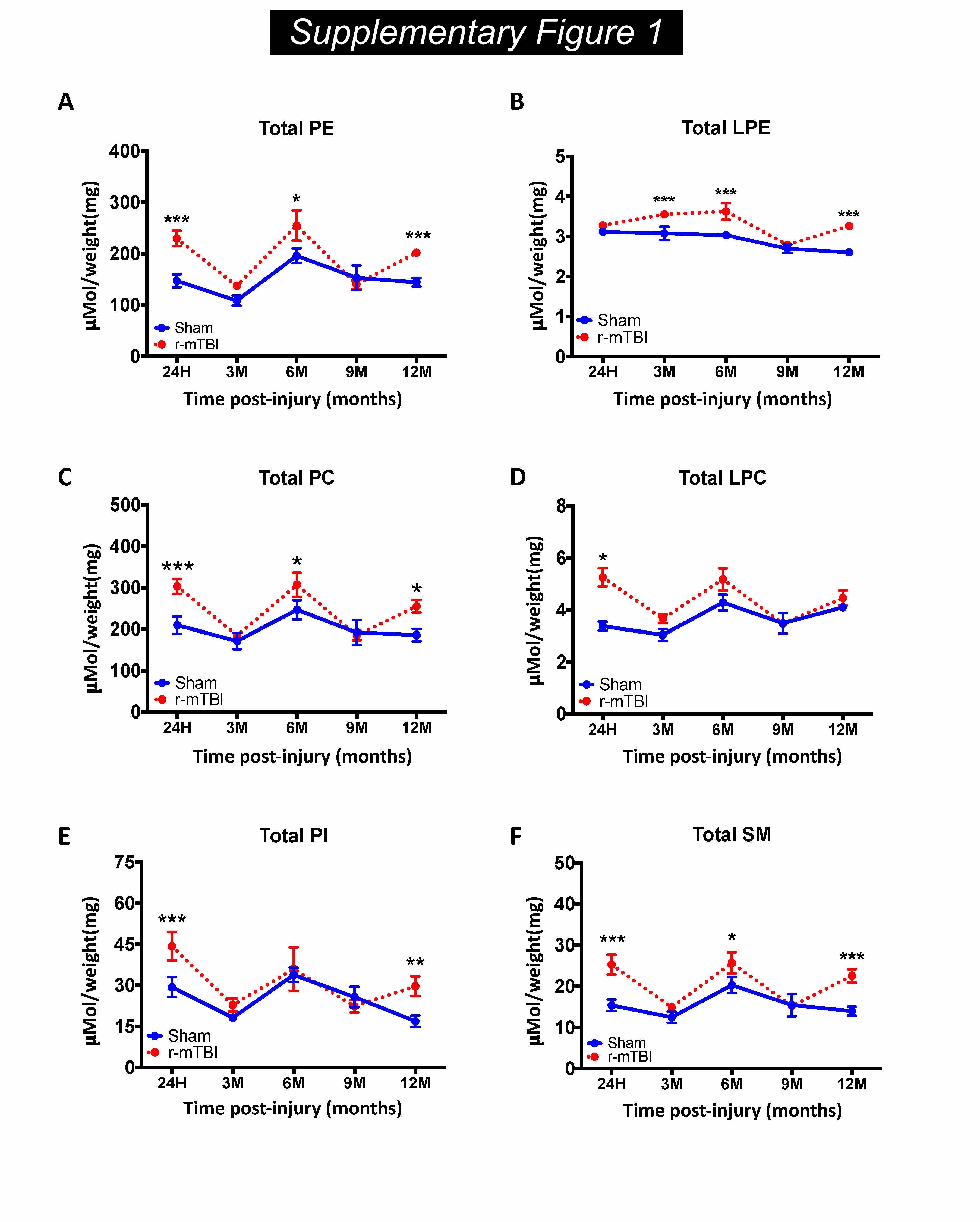

Supplement: FIGURE S1 — Total Phospholipid levels in the hippocampi of r-mTBI mice. Significant changes in total phospholipid (PE, LPE, PC, LPC, PI, SM) species in the hippocampi of a mouse model of repetitive-mTBI (A–F). N = 4 for all groups at each time point. All data represent mean μM per (5.5 mg) wet weight ± SEM. Individual molecular lipid species were quantified by LC/MS and were summed after LipidomeDB analyses to generate total phospholipid levels. Asterisks represent ∗P < 0.05; ∗∗P < 0.01; ∗∗∗P < 0.001 for comparisons between sham/r-mTBI mice. PE, Phosphatidylethanolamine; LPE, Lysophosphatidylethanolamine; PC, Phosphatidylcholine; LPC, Lysophosphatidylcholine; PI, Phosphatidylinositol; SM, Sphingomyelin. [file Image_9.JPEG]

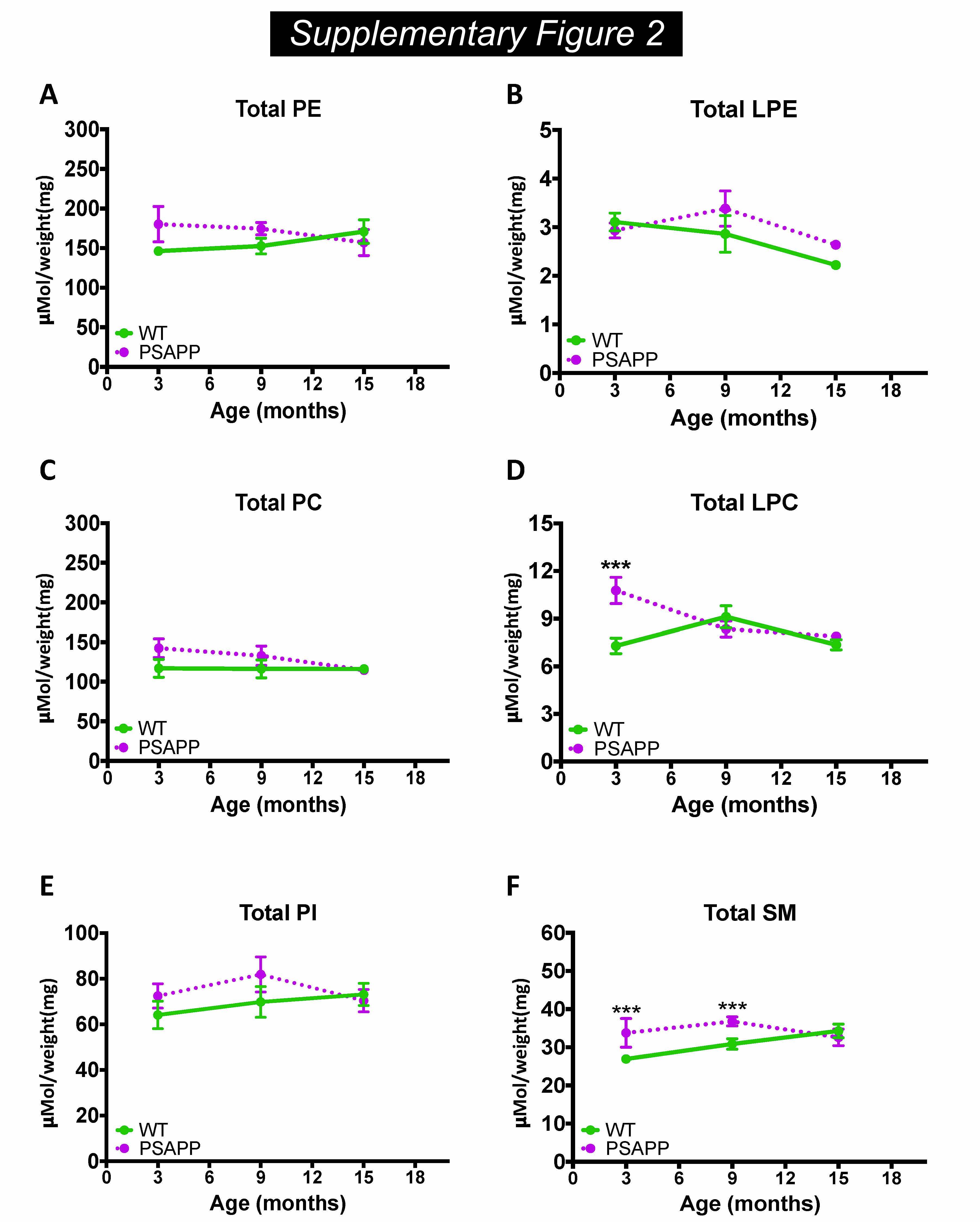

Supplement: FIGURE S2 — Total Phospholipid levels in the hippocampi of PSAPP mice. Significant changes in total phospholipid (PE, LPE, PC, LPC, PI, SM) species in the hippocampi of the PSAPP mouse model compared to littermate controls (A–F). N = 4 for all groups at each time point. All data represents mean μM per (5.5 mg) wet weight ± SEM. Individual molecular lipid species were quantified by LC/MS and were summed after LipidomeDB analyses to generate total phospholipid levels. Asterisks represent ∗∗∗P < 0.001 for comparisons between PSAPP/WT mice. PE, Phosphatidylethanolamine; LPE, Lysophosphatidylethanolamine; PC, Phosphatidylcholine; LPC, Lysophosphatidylcholine; PI, Phosphatidylinositol; SM, Sphingomyelin. [file Image_10.JPEG]

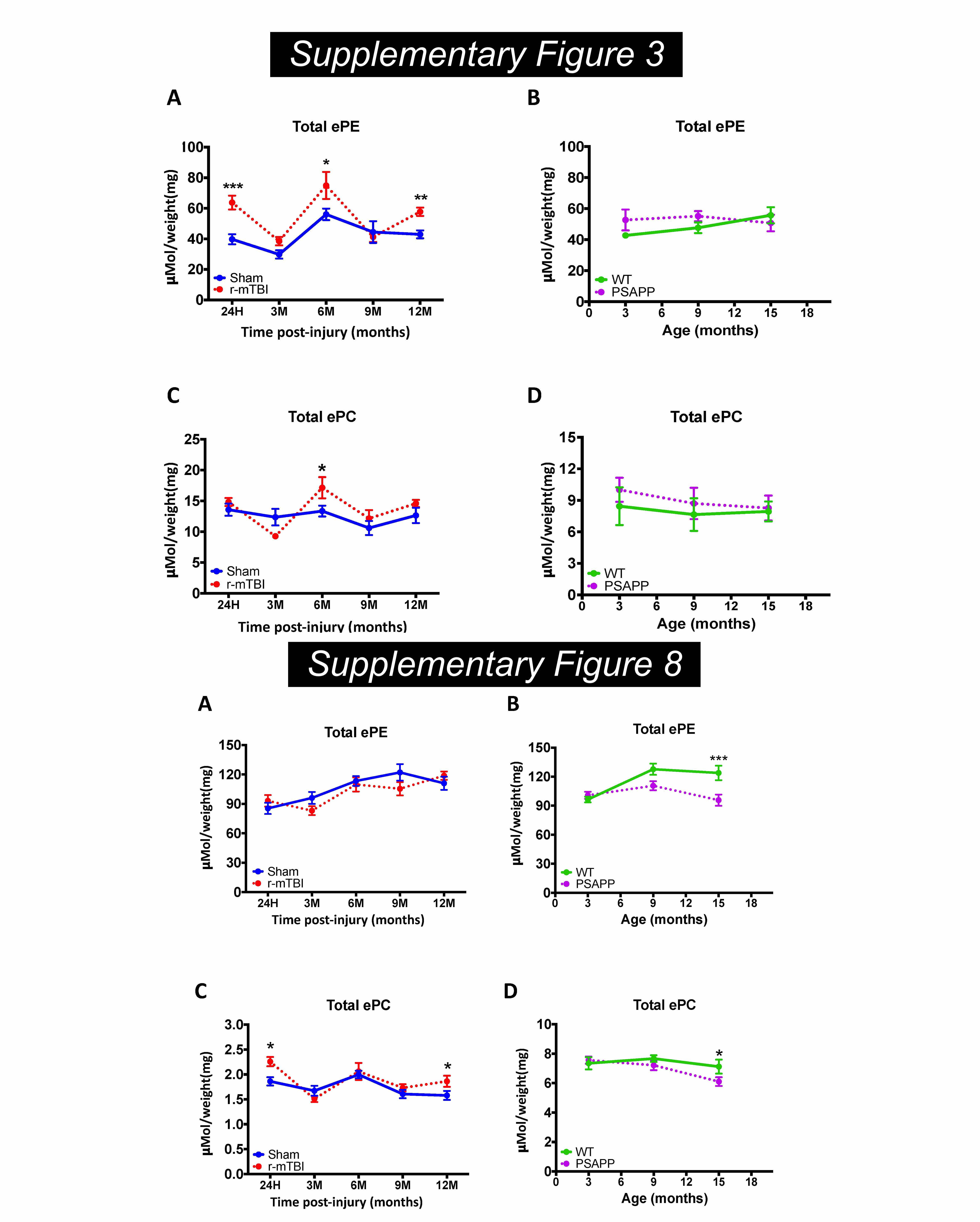

Supplement: FIGURE S3 — Total etherPE and ether PC levels in the hippocampus of repetitive-mTBI and PSAPP mice. Significant changes in total etherPE and etherPC lipid species in the hippocampus of a mouse model of repetitive-mTBI and AD (A–D). Sample size for all groups across all time points is n = 4. All data represents mean μM per (5.5 mg) wet weight ± SEM. Individual molecular lipid species were quantified by LC/MS and were summed after LipidomeDB analyses to generate total etherphospholipid levels. Asterisks represents ∗P < 0.05; ∗∗P < 0.01; ∗∗∗P < 0.001 for comparisons between sham/r-mTBI mice or PSAPP/WT mice. ePE, etherphosphatidylethanolamine; ePC, etherphosphatidylcholine. [file Image_11.JPEG]

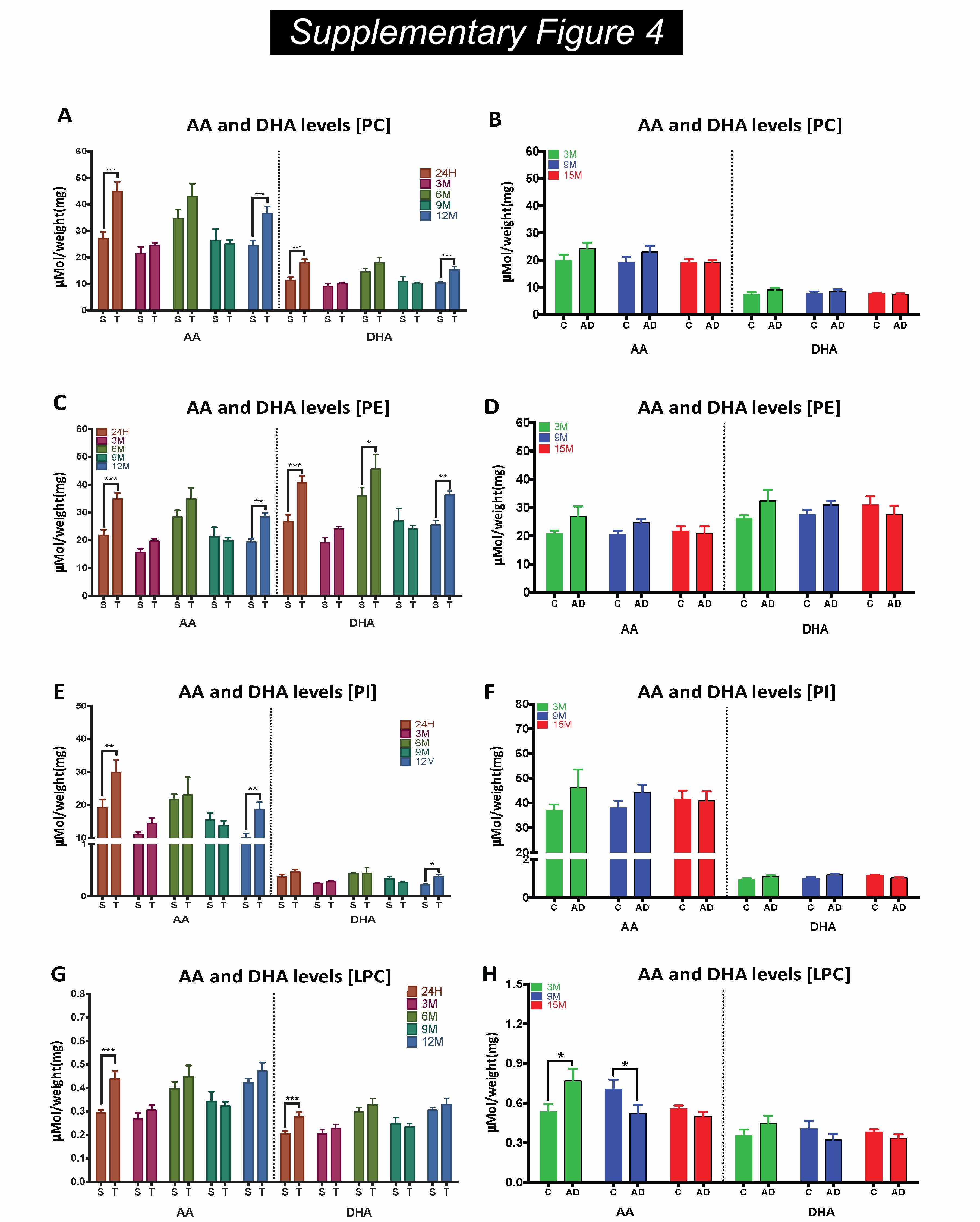

Supplement: FIGURE S4 — Arachidonic acid and decosahexaenoic acid containing phospholipid species in the hippocampus of repetitive mTBI and PSAPP mice. Significant changes in arachidonic acid and decosahexaenoic acid containing PC, PE and PI species in the hippocampus of a mouse model of repetitive-mTBI and AD (A–H). Sample size for all groups across all time points is n = 4. Data represents mean μmol per wet weight (5.5 mg) ± SEM. Individual molecular lipid species were quantified by LC/MS and were summed after LipidomeDB analyses to generate arachidonic and decosahexaenoic acid levels for each phospholipid species. Asterisks represents ∗P < 0.05; ∗∗P < 0.01; ∗∗∗P < 0.001 for comparisons between sham/r-mTBI mice or WT/PSAPP mice. PE, Phosphatidylethanolamine; PC, Phosphatidylcholine; PI, Phosphatidylinositol; LPC, Lysophosphatidylcholine; AA, arachidonic acid; DHA, decosahexaenoic acid. [file Image_1.JPEG]

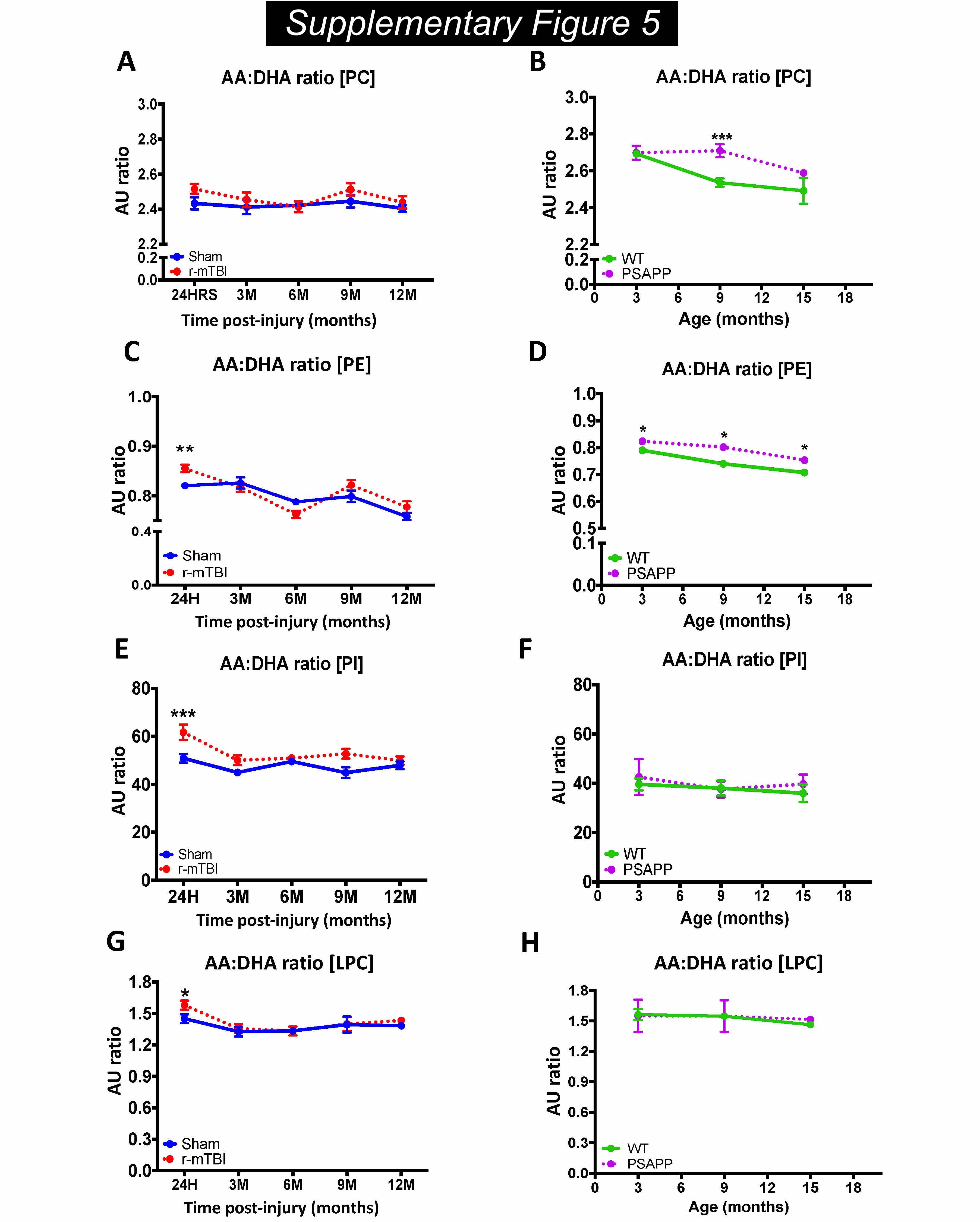

Supplement: FIGURE S5 — Arachidonic acid to decosahexaenoic acid ratio containing phospholipid species in the hippocampus of repetitive mTBI and PSAPP mice. Significant changes in arachidonic acid to decosahexaenoic acid ratio for PC, PE, and PI phospholipid species in the hippocampus of a mouse model of repetitive-mTBI and AD (A–H). Sample size for all groups across all time points is n = 4. Data represents arbitrary value (ratio) value ± SEM. Individual molecular lipid species were quantified by LC/MS and were summed after LipidomeDB analyses to generate AA and DHA levels for each phospholipid species, and a ratio of AA to DHA was compiled from these values. Asterisks represents ∗P < 0.05; ∗∗P < 0.01; ∗∗∗P < 0.001 for comparisons between sham/r-mTBI mice or WT/PSAPP mice. PE, Phosphatidylethanolamine; PC, Phosphatidylcholine; PI, Phosphatidylinositol; LPC, Lysophosphatidylcholine; AA, arachidonic acid; DHA, decosahexaenoic acid. [file Image_12.JPEG]

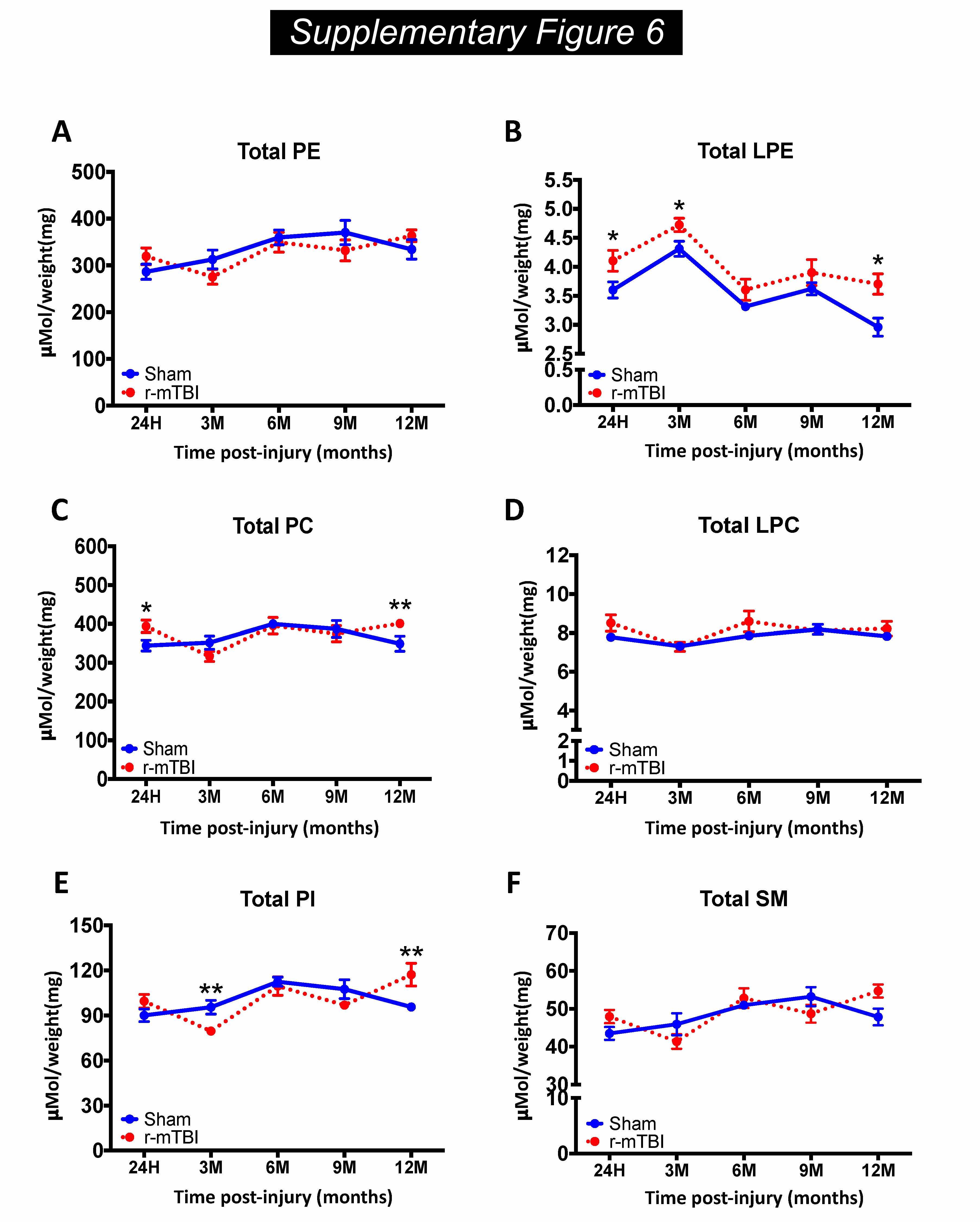

Supplement: FIGURE S6 — Total Phospholipid levels in the cortex of r-mTBI mice. Significant changes in total phospholipid (PE, LPE, PC, LPC, PI, SM) species in the cortex of a mouse model of repetitive-mTBI (A–F). Sample size for all groups across all time points is n = 4. All data represents mean μM per (10) wet weight ± SEM. Individual molecular lipid species were quantified by liquid chromatography/mass spectrometry and were summed after LipidomeDB analyses to generate total phospholipid levels. Asterisks represents ∗P < 0.05; ∗∗P < 0.01; ∗∗∗P < 0.001 for comparisons between sham/r-mTBI mice. PE, Phosphatidylethanolamine; LPE, Lysophosphatidylethanolamine; PC, Phosphatidylcholine; LPC, Lysophosphatidylcholine; PI, Phosphatidylinositol; SM, Sphingomyelin. [file Image_13.JPEG]

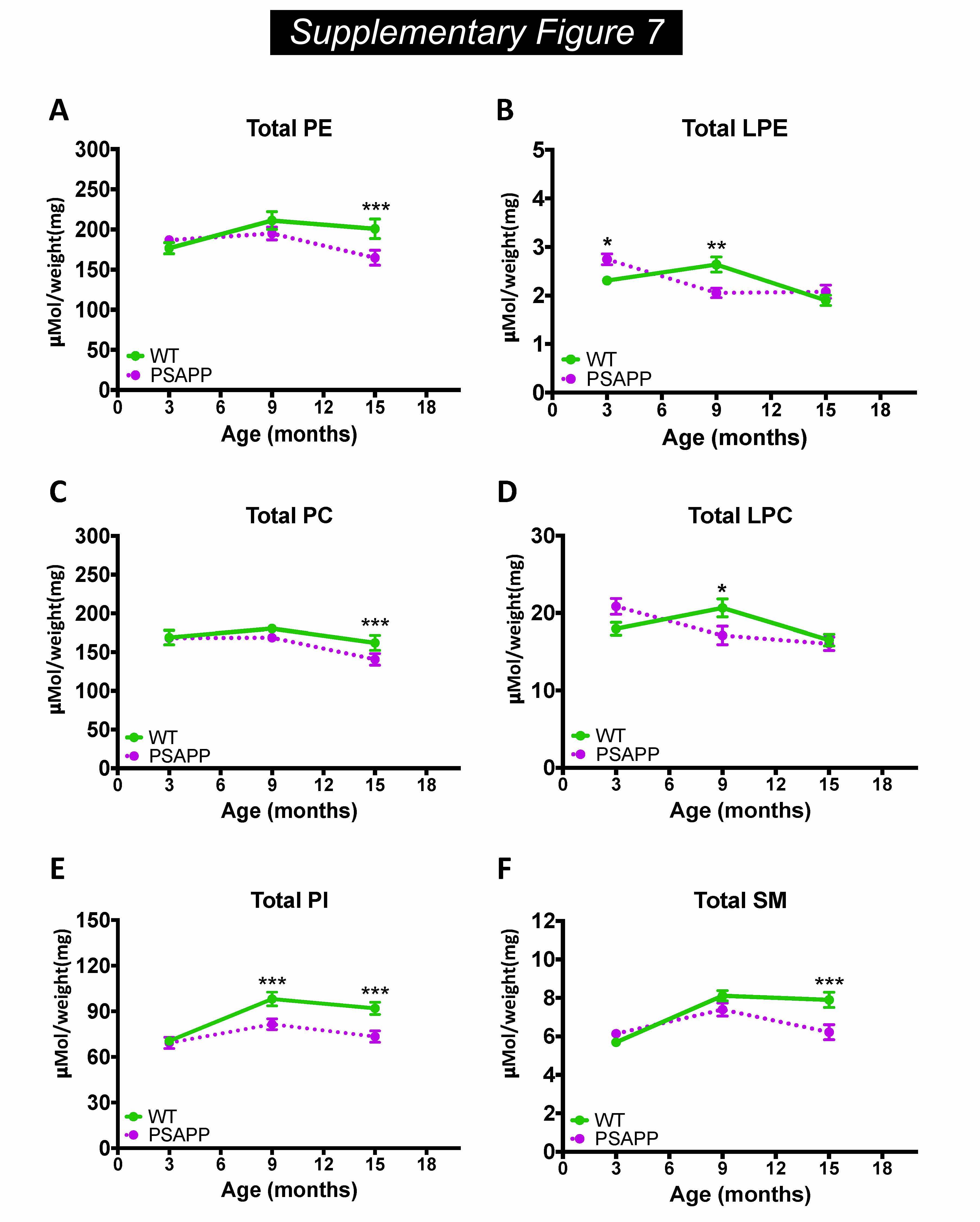

Supplement: FIGURE S7 — Total Phospholipid levels in the cortex of PSAPP mice. Significant changes in total phospholipid (PE, LPE, PC, LPC, PI, SM) species in the cortex of a PSAPP mouse model (A–F). Sample size for all groups across all time points is n = 4. All data represents mean μM per (10 mg) wet weight ± SEM. Individual molecular lipid species were quantified by liquid chromatography/mass spectrometry and were summed after LipidomeDB analyses to generate total phospholipid levels. Asterisks represents ∗∗∗P < 0.001 for comparisons between PSAPP/WT mice. PE, Phosphatidylethanolamine; LPE, Lysophosphatidylethanolamine; PC, Phosphatidylcholine; LPC, Lysophosphatidylcholine; PI, Phosphatidylinositol; SM, Sphingomyelin. [file Image_14.JPEG]

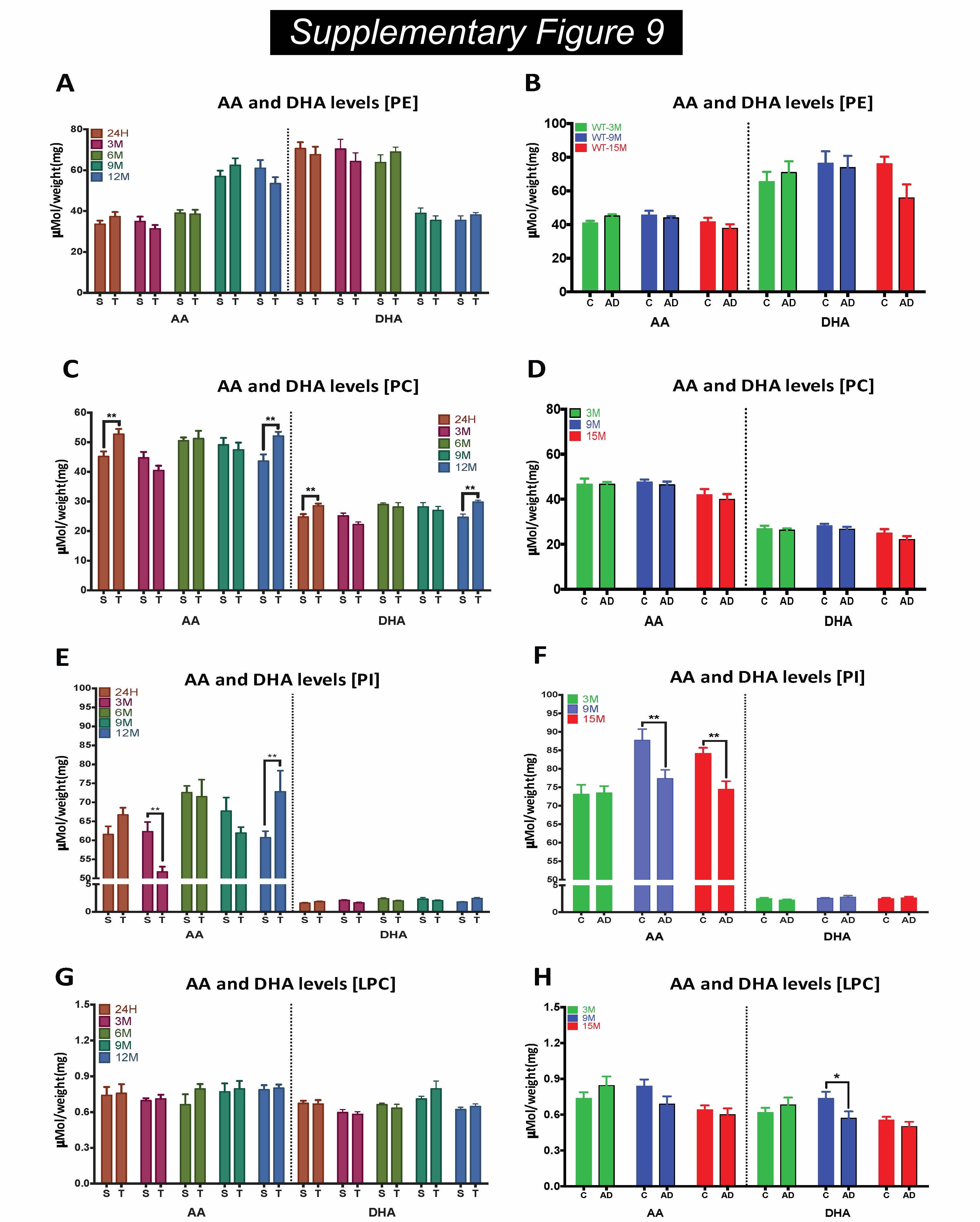

Supplement: FIGURE S9 — Arachidonic acid and decosahexaenoic acid containing phospholipid species in the cortex of repetitive mTBI and PSAPP mice. Significant changes in arachidonic acid and decosahexaenoic acid containing PC (C,D), PE (A,B), PI (E,F), and LPC (G,H) species in the cortex of a mouse model of repetitive-mTBI and AD. Sample size for all groups across all time points is n = 4. Data represents mean μmol per wet weight (10 mg) ± SEM. Individual molecular lipid species were quantified by liquid chromatography/mass spectrometry and were summed after LipidomeDB analyses to generate arachidonic and decosahexaenoic acid levels for each phospholipid species. Asterisks represents ∗∗P < 0.01 for comparisons between sham/r-mTBI mice or WT/PSAPP mice. PE, Phosphatidylethanolamine; PC, Phosphatidylcholine; PI, Phosphatidylinositol; LPC, Lysophosphatidylcholine; AA, arachidonic acid; DHA, decosahexaenoic acid. [file Image_2.JPEG]

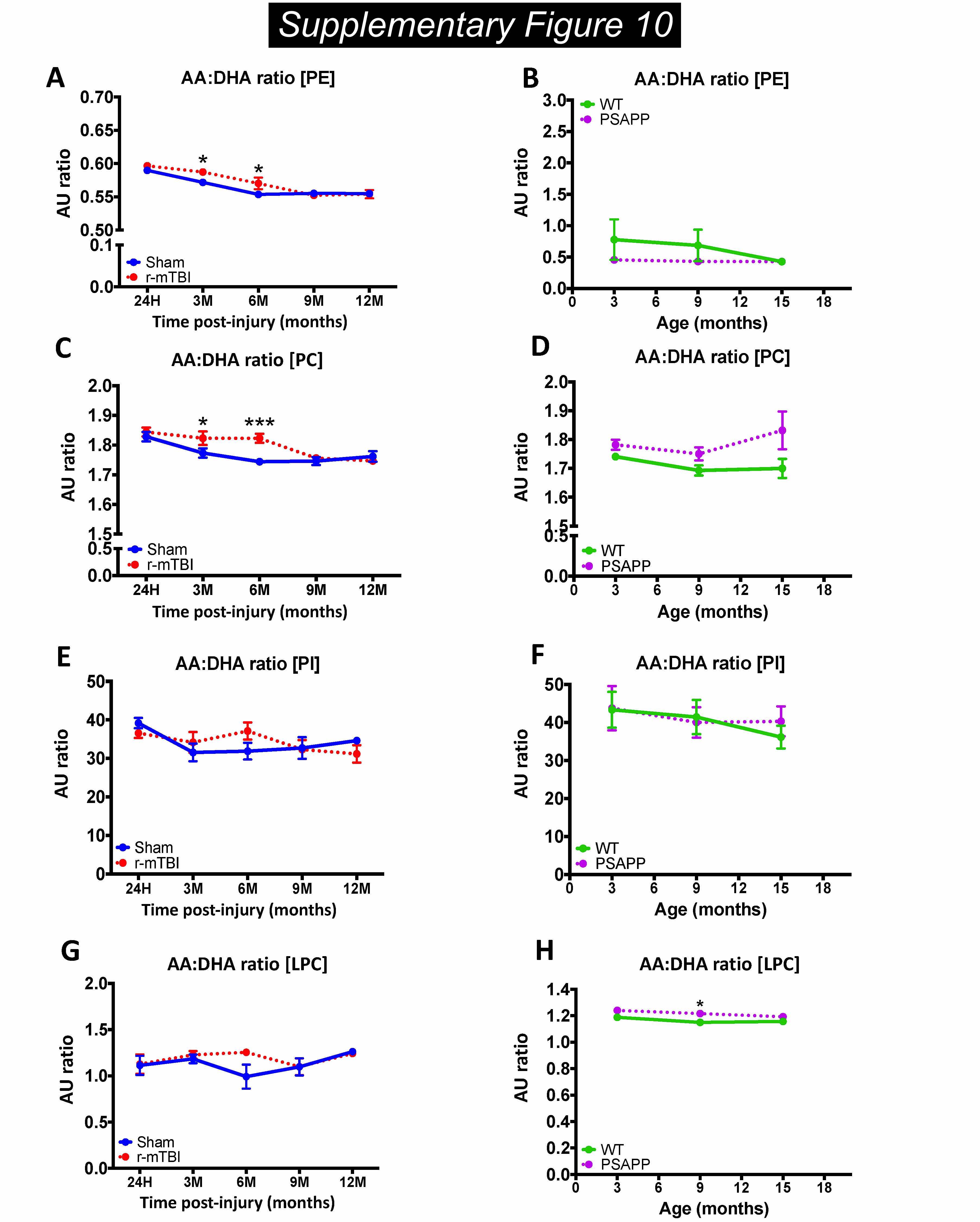

Supplement: FIGURE S10 — Arachidonic acid to decosahexaenoic acid ratio containing phospholipid species in the cortex of repetitive mTBI and PSAPP mice. Significant changes in arachidonic acid to decosahexaenoic acid ratio for PC (C,D), PE (A,B), PI (E,F), and LPC (G,H) phospholipid species in the cortex of a mouse model of repetitive-mTBI and AD. Sample size for all groups across all time points is n = 4. Data represents arbitrary value (ratio) value ± SEM. Individual molecular lipid species were quantified by liquid chromatography/mass spectrometry and were summed after LipidomeDB analyses to generate AA and DHA levels for each phospholipid species, and a ratio of AA to DHA was compiled from these values. Asterisks represents ∗P < 0.05; ∗∗∗P < 0.001 for comparisons between sham/r-mTBI mice or WT/PSAPP mice. PE, Phosphatidylethanolamine; PC, Phosphatidylcholine; PI, Phosphatidylinositol; LPC, Lysophosphatidylcholine; AA, arachidonic acid; DHA, decosahexaenoic acid. [file Image_15.JPEG]

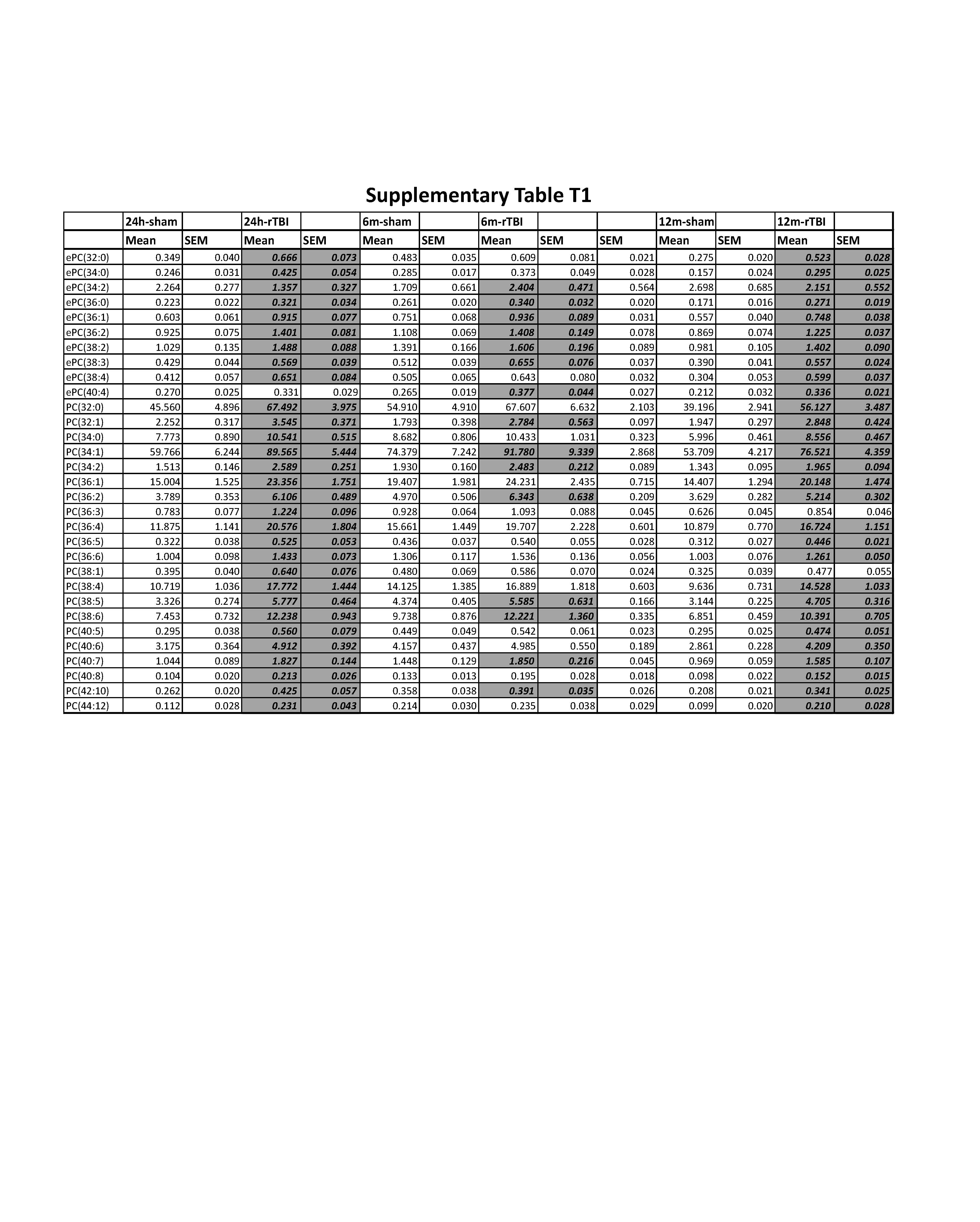

Supplement: TABLE 1 — Phosphatidylcholine lipid species in the hippocampus of r-mTBI mice. Significant changes in individual species containing phosphatidylcholine. Sample size for all groups across all time points is n = 4. All data represents mean μM per (5.5 mg) wet weight ± SEM. Individual molecular lipid species were quantified by liquid chromatography/mass spectrometry. Highlighted boxes show significantly regulated levels (P < 0.001) between repetitive-mTBI/sham mice based on mixed linear modeling regression analysis. [file Image_3.JPEG]

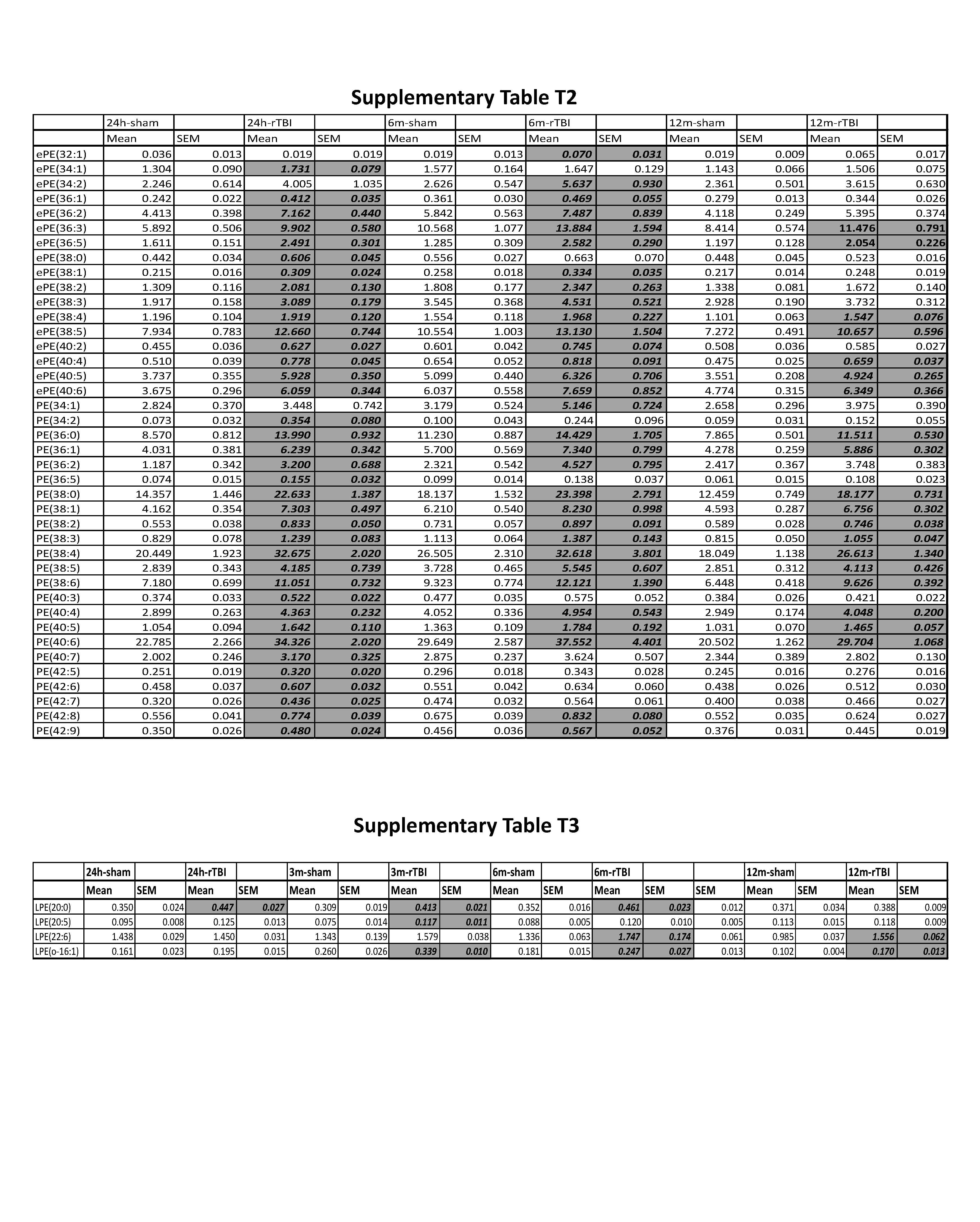

Supplement: TABLE 2 — Phosphatidylethanolamine lipid species in the hippocampus of r-mTBI mice. Significant changes in individual species containing phosphatidylethanolamine. Sample size for all groups across all time points is n = 4. All data represents mean μM per (5.5 mg) wet weight ± SEM. Individual molecular lipid species were quantified by liquid chromatography/mass spectrometry. Highlighted boxes show significantly regulated levels (P < 0.001) between repetitive-mTBI/sham mice based on mixed linear modeling regression analysis. [file Image_4.JPEG]

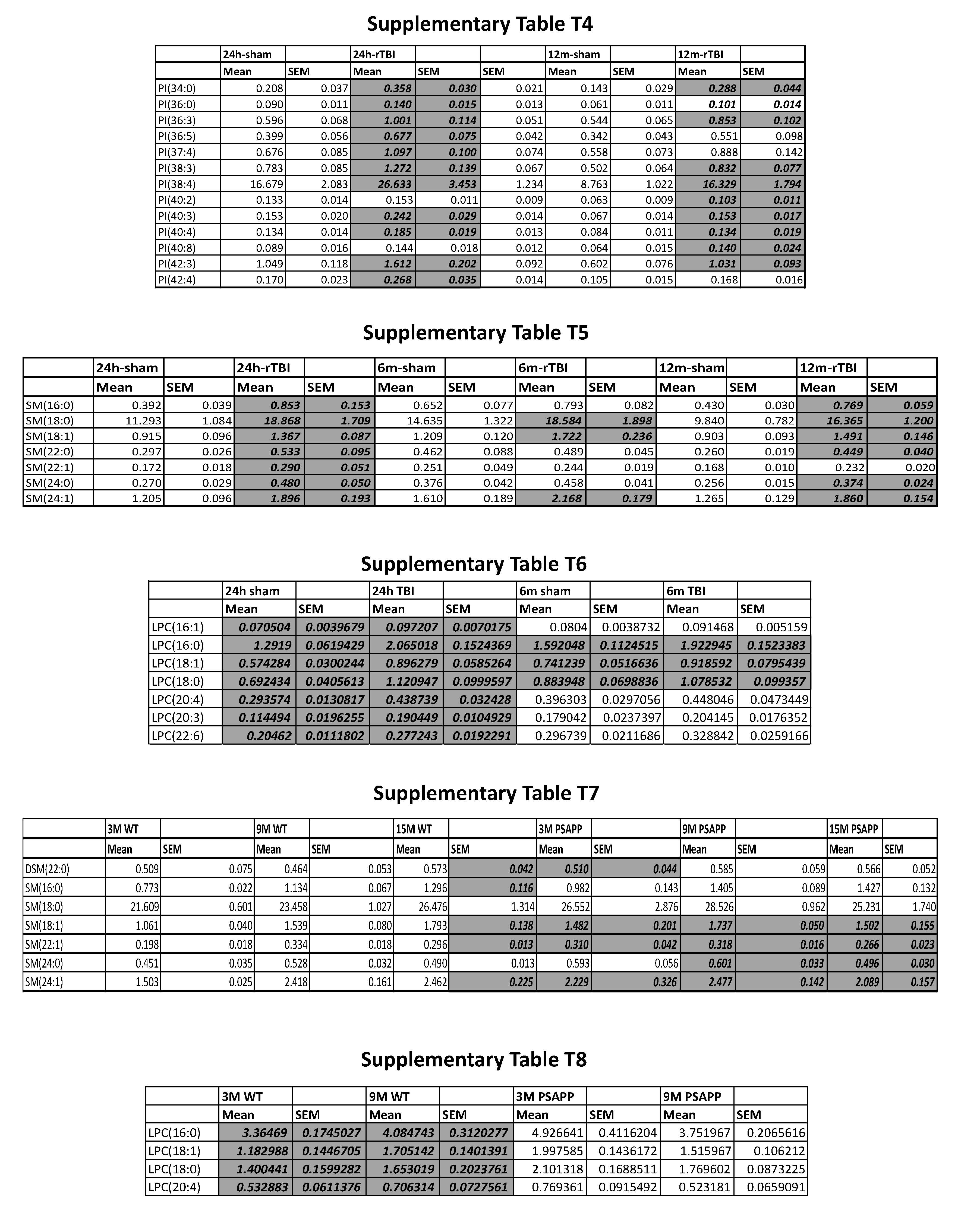

Supplement: TABLE 4 — Phosphatidylinositol lipid species in the hippocampus of r-mTBI mice. Significant changes in individual species containing phosphatidylinositiol. Sample size for all groups across all time points is n = 4. All data represents mean μM per (5.5 mg) wet weight ± SEM. Individual molecular lipid species were quantified by liquid chromatography/mass spectrometry. Highlighted boxes show significantly regulated levels (P < 0.001) between repetitive-mTBI/sham mice based on mixed linear modeling regression analysis. [file Image_5.JPEG]

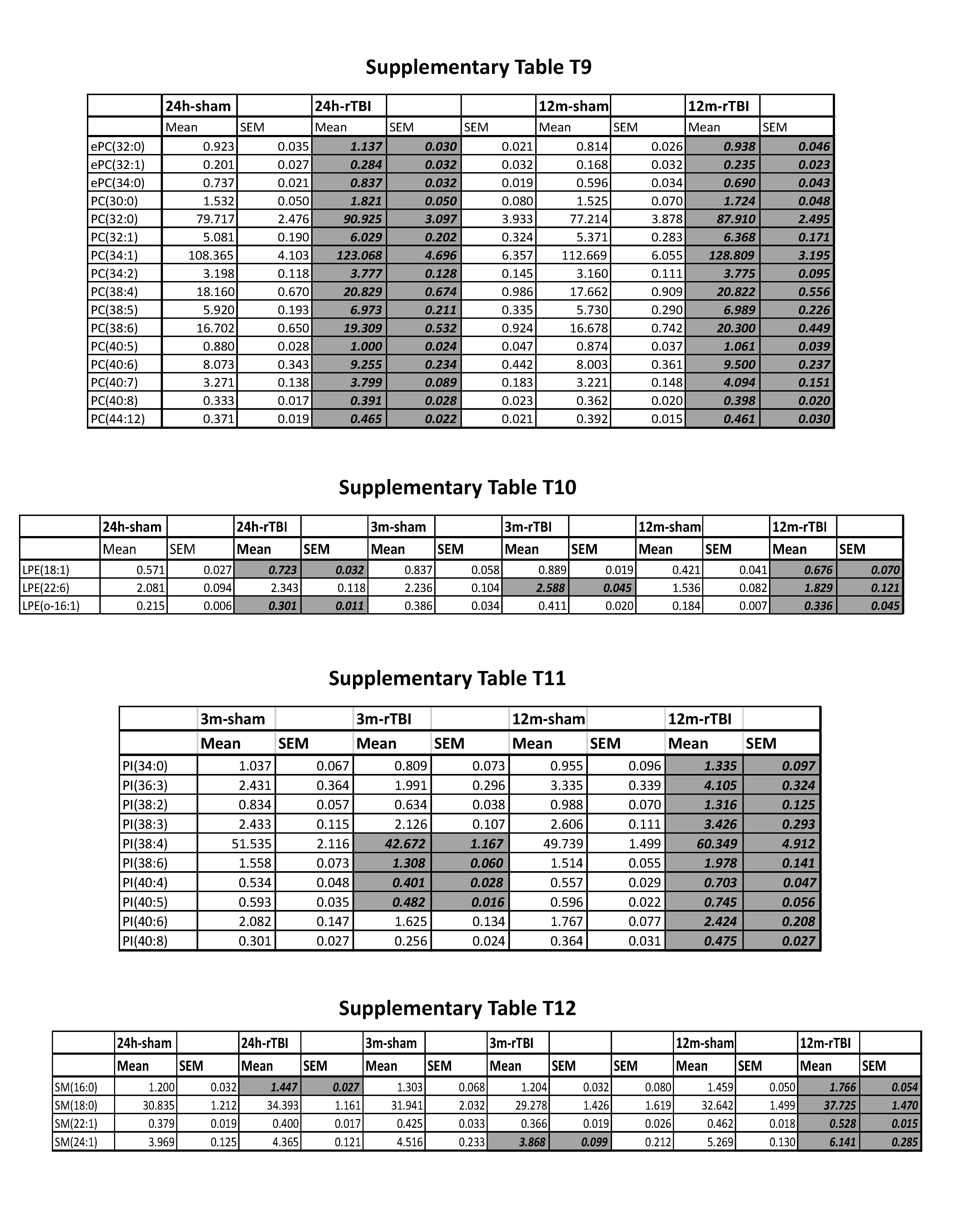

Supplement: TABLE 9 — Phosphatidylcholine lipid species in the cortex of r-mTBI mice. Significant changes in individual species containing phosphatidylcholine. Sample size for all groups across all time points is n = 4. All data represents mean μM per (10 mg) wet weight ± SEM. Individual molecular lipid species were quantified by liquid chromatography/mass spectrometry. Highlighted boxes show significantly regulated levels (P < 0.001) between repetitive-mTBI/sham mice based on mixed linear modeling regression analysis. [file Image_6.JPEG]

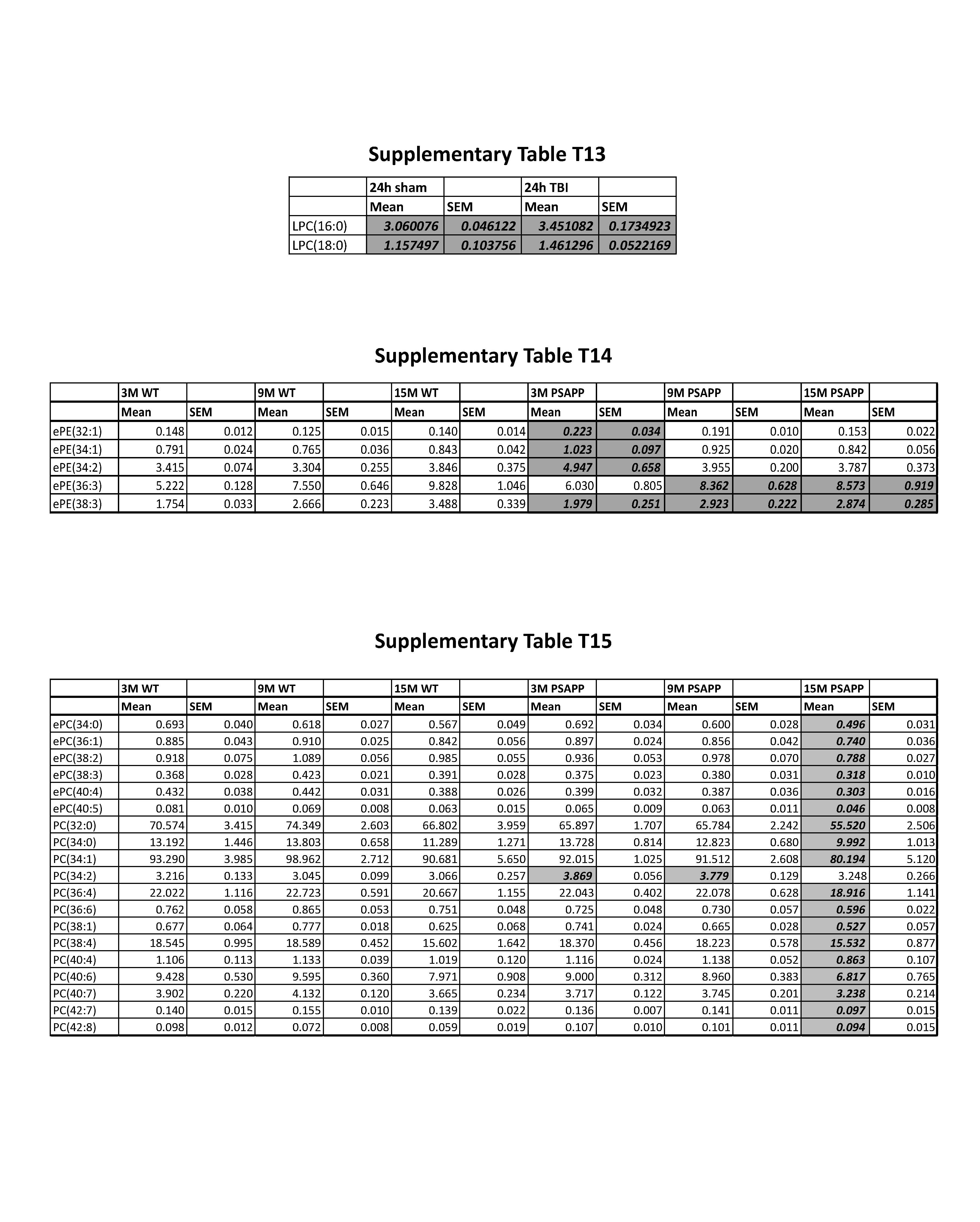

Supplement: TABLE 13 — Lysophosphatidycholine lipid species in the cortex of r-mTBI mice. Significant changes in individual species containing lysophosphatidylcholine. Sample size for all groups across all time points is n = 4. All data represents mean μM per (10 mg) wet weight ± SEM. Individual molecular lipid species were quantified by liquid chromatography/mass spectrometry. Highlighted boxes show significantly regulated levels (P < 0.001) between repetitive-mTBI/sham mice based on mixed linear modeling regression analysis. [file Image_7.JPEG]

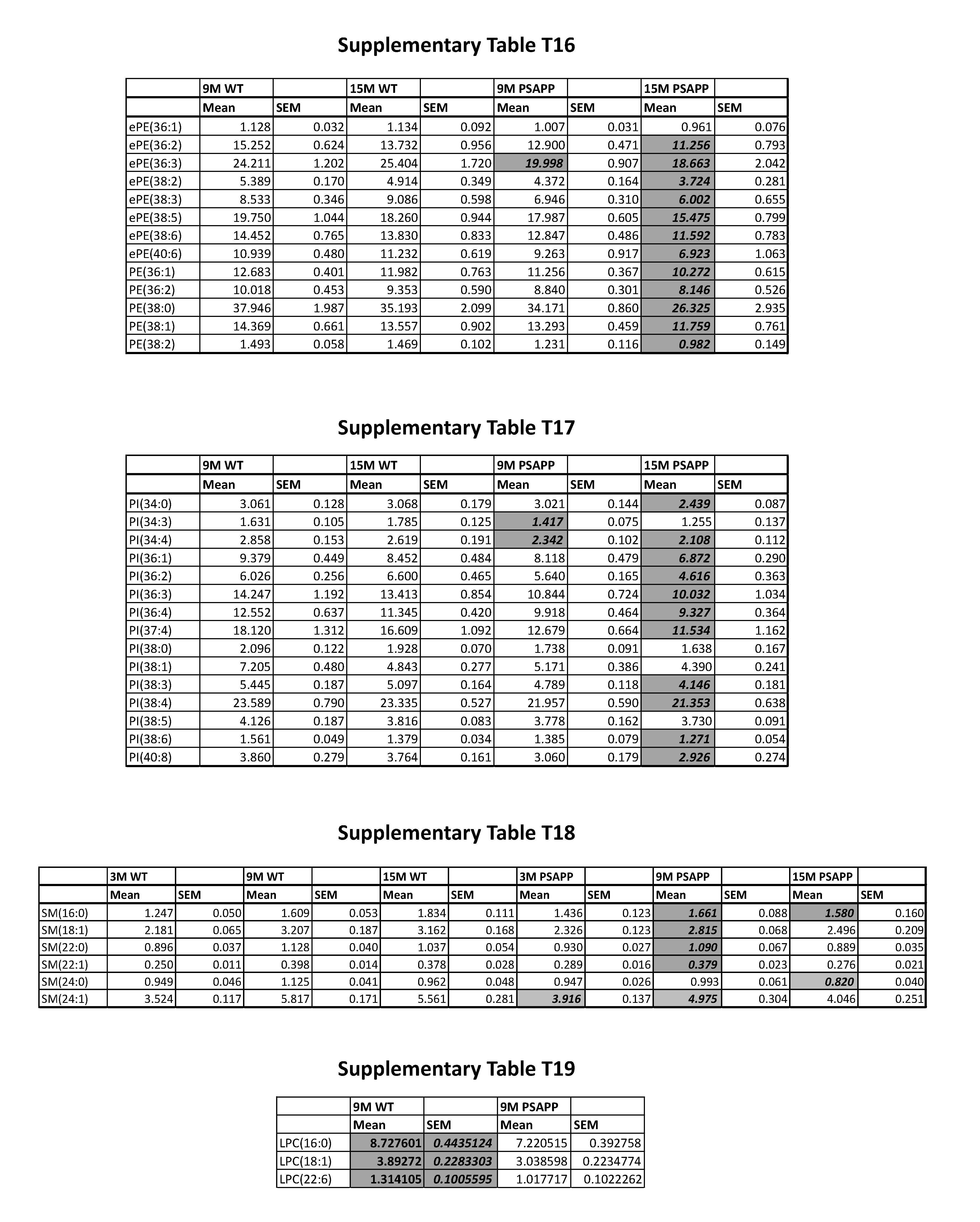

Supplement: TABLE 16 — Phosphatidylethanolamine lipid species in the cortex of PSAPP mice. Significant changes in individual species containing phosphatidylethanolamine. Sample size for all groups across all time points is n = 4. All data represents mean μM per (10 mg) wet weight ± SEM. Individual molecular lipid species were quantified by liquid chromatography/mass spectrometry. Highlighted boxes show significantly regulated levels (P < 0.001) between PSAPP/WT mice based on mixed linear modeling regression analysis. [file Image_8.JPEG]
